# Ancient and recent introgression shape the evolutionary history of pollinator adaptation and speciation in a model monkeyflower radiation (*Mimulus* section *Erythranthe*)

**DOI:** 10.1371/journal.pgen.1009095

**Published:** 2021-02-22

**Authors:** Thomas C. Nelson, Angela M. Stathos, Daniel D. Vanderpool, Findley R. Finseth, Yao-wu Yuan, Lila Fishman

**Affiliations:** 1 Division of Biological Sciences, University of Montana, Missoula, Montana, United States of America; 2 Department of Ecology and Evolutionary Biology, University of Connecticut, Storrs, Connecticut, United States of America; University of Wyoming, UNITED STATES

## Abstract

Inferences about past processes of adaptation and speciation require a gene-scale and genome-wide understanding of the evolutionary history of diverging taxa. In this study, we use genome-wide capture of nuclear gene sequences, plus skimming of organellar sequences, to investigate the phylogenomics of monkeyflowers in *Mimulus* section *Erythranthe* (27 accessions from seven species*)*. Taxa within *Erythranthe*, particularly the parapatric and putatively sister species *M*. *lewisii* (bee-pollinated) and *M*. *cardinalis* (hummingbird-pollinated), have been a model system for investigating the ecological genetics of speciation and adaptation for over five decades. Across >8000 nuclear loci, multiple methods resolve a predominant species tree in which *M*. *cardinalis* groups with other hummingbird-pollinated taxa (37% of gene trees), rather than being sister to *M*. *lewisii* (32% of gene trees). We independently corroborate a single evolution of hummingbird pollination syndrome in *Erythranthe* by demonstrating functional redundancy in genetic complementation tests of floral traits in hybrids; together, these analyses overturn a textbook case of pollination-syndrome convergence. Strong asymmetries in allele sharing (Patterson’s D-statistic and related tests) indicate that gene tree discordance reflects ancient and recent introgression rather than incomplete lineage sorting. Consistent with abundant introgression blurring the history of divergence, low-recombination and adaptation-associated regions support the new species tree, while high-recombination regions generate phylogenetic evidence for sister status for *M*. *lewisii* and *M*. *cardinalis*. Population-level sampling of core taxa also revealed two instances of chloroplast capture, with Sierran *M*. *lewisii* and Southern Californian *M*. *parishii* each carrying organelle genomes nested within respective sympatric *M*. *cardinalis* clades. A recent organellar transfer from *M*. *cardinalis*, an outcrosser where selfish cytonuclear dynamics are more likely, may account for the unexpected cytoplasmic male sterility effects of selfer *M*. *parishii* organelles in hybrids with *M*. *lewisii*. Overall, our phylogenomic results reveal extensive reticulation throughout the evolutionary history of a classic monkeyflower radiation, suggesting that natural selection (re-)assembles and maintains species-diagnostic traits and barriers in the face of gene flow. Our findings further underline the challenges, even in reproductively isolated species, in distinguishing re-use of adaptive alleles from true convergence and emphasize the value of a phylogenomic framework for reconstructing the evolutionary genetics of adaptation and speciation.

## Author summary

Adaptive radiations, which involve both divergent evolution of new traits and recurrent trait evolution, provide insight into the processes that generate and maintain organismal diversity. However, rapid radiations also generate particular challenges for inferring the evolutionary history and mechanistic basis of adaptation and speciation, as multiple processes can cause different parts of the genome to have distinct phylogenetic trees. Thus, inferences about the mode and timing of divergence and the causes of parallel trait evolution require a fine-grained understanding of the flow of genomic variation through time. In this study, we used genome-wide sampling of thousands of genes to re-construct the evolutionary histories of a model plant radiation, the monkeyflowers of *Mimulus* section *Erythranthe*. Work over the past half-century has established the parapatric and putatively sister species *M*. *lewisii* (bee-pollinated) and *M*. *cardinalis* (hummingbird-pollinated, as are three other species in the section) as textbook examples of both rapid speciation via shifts in pollination syndrome and convergent evolution of floral syndromes. Our phylogenomic analyses re-write both of these stories, placing *M*. *cardinalis* in a clade with other hummingbird-pollinated taxa and demonstrating that abundant introgression between ancestral lineages as well as in areas of current sympatry contributes to the real (but misleading) affinities between *M*. *cardinalis* and *M*. *lewisii*. This work illustrates the pervasive influence of gene flow and introgression during adaptive radiation and speciation, and underlines the necessity of a gene-scale and genome-wide phylogenomics framework for understanding trait divergence, even among well-established species.

## Introduction

Adaptive radiations are engines of biodiversity and thus natural laboratories for understanding its origins [1–5]. During radiations, natural selection can cause both phenotypic divergence as populations move into novel environments and convergence when different populations adapt to similar ecological conditions [6, 7]. Divergence provides the opportunity to re-construct the ecological context and genetic basis of adaptive walks, while repeated evolution can reveal the importance of genetic vs. environmental constraints in shaping convergent phenotypes (reviewed in [8]). Furthermore, the processes of adaptation and speciation are tightly intertwined in radiations, and recent radiations help reveal the processes and genes underlying lineage diversification [8–11]. A strong phylogenetic framework is necessary both for understanding the process of speciation and for tracing phenotypic evolution across species (e.g. inferring convergence vs. a single mutational origin for similar phenotypes) [12]. However, the rapid diversification characteristic of adaptive radiations also confounds definition of a single "species tree" [13]. Thus, understanding adaptation and speciation within radiations requires a phylogenomic context that captures the diversity of evolutionary histories across recently diverged genomes [4, 14, 15].

Two processes confound the reconstruction of a universal genome-wide "species tree", while also affecting the course of adaptation and speciation [16]. Incomplete lineage sorting (ILS), in which different lineages randomly sample the same alleles polymorphic in their ancestor, can persist after rapid splitting of ancestral populations [17]. In addition, incomplete reproductive isolation between incipient species in areas of sympatry may allow gene flow and introgression that lead to further discordance between the genealogical relationships at any one locus and the deeper species relationships. Both ILS and introgression complicate the inference of species trees, but they have very different impacts on the processes of adaptation. In particular, introgression may cause adaptive alleles, and thus the traits they confer, to be shared among species that are not otherwise closely related [11, 18]. Conversely, hybridizing species that are not closely related may appear as sister taxa in phylogenies strongly influenced by introgressed loci (whether those loci are adaptive or not). Such introgression is empirically common, as evidenced by sharp discordance between nuclear and organellar (mitochondrial, chloroplast) phylogenetic trees in many plants [19]and animals [20]. Thus, disentangling the contributions of ILS and introgression to the flow of genetic variation through radiations is important not only to properly characterize the historical process of adaptive evolution, but to reveal its mechanisms. Applying phylogenomic approaches across entire radiations can provide nuanced insight into the constraints, causes, and consequences of adaptive evolution, as well as the processes that structure sequence evolution across complex genomes.

**Fig 1:**
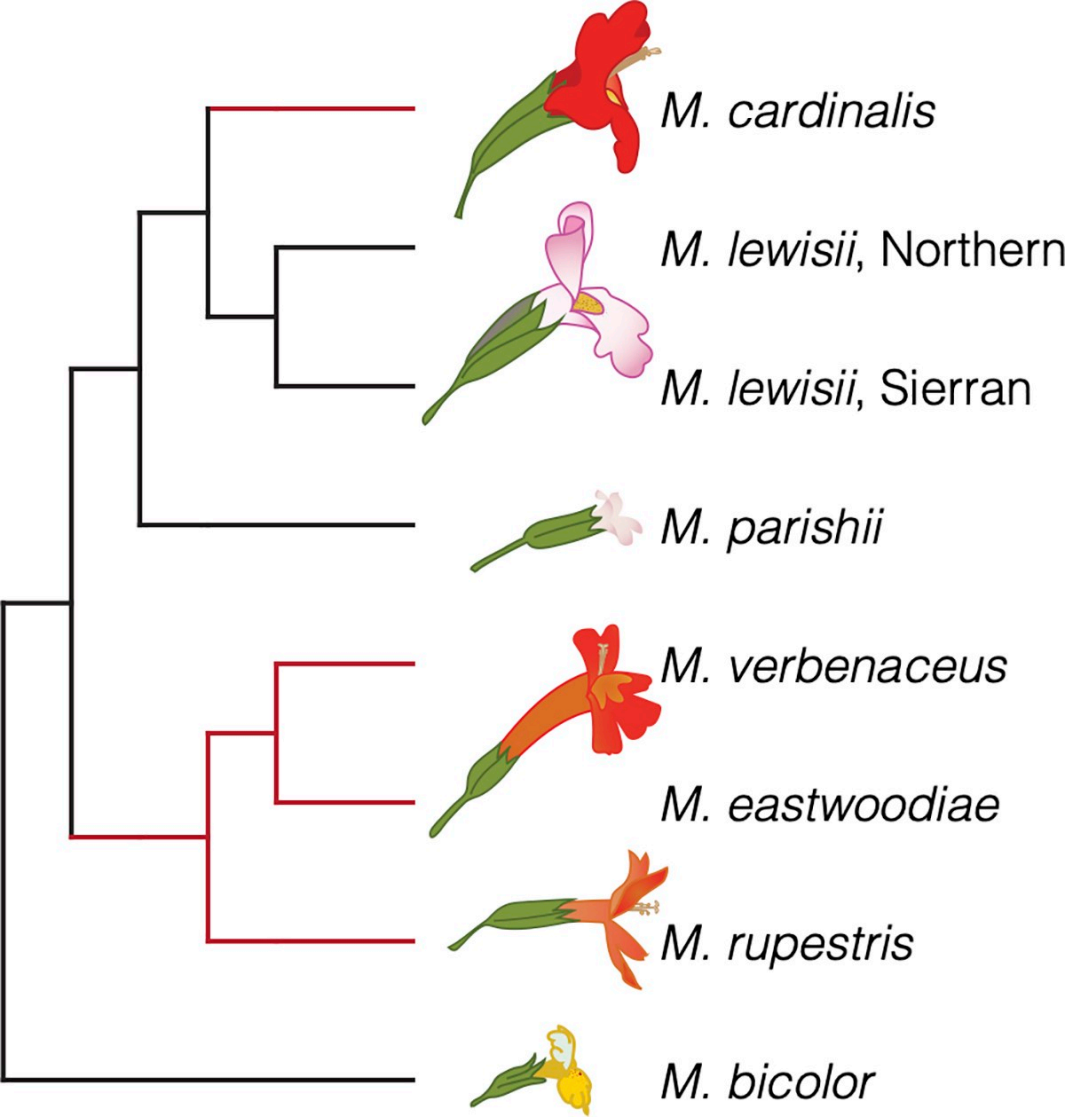
*Mimulus* section *Erythranthe*, with *M*. *bicolor* as an outgroup, as defined by previous phylogenetic treatments [21, 22, 40]. The two putative derivations of hummingbird pollination shown in red. Branch lengths are arbitrary and chosen for visual clarity.

Here, we present phylogenomic re-assessment of the evolutionary history of a classic adaptive radiation in flowering plants, the monkeyflowers of *Mimulus* section *Erythranthe* (Phrymaceae) [21, 22]. Recent taxonomic re-organizations of monkeyflowers have re-named many *Mimulus*, including these taxa, as genus *Erythranthe* [23], and have also split several species within this section [24]. However, in the absence of a well-resolved family-level phylogeny, and for consistency with previous work, we refer to these taxa as *Mimulus* section *Erythranthe* and retain previous species names [22]. The *Erythranthe* section contains five taxa with flowers adapted for hummingbird pollination (narrow red corolla tubes with little or no landing pad for bees, often abundant nectar; Fig 1). *Mimulus cardinalis* is common in riparian habitats across a broad latitudinal range in western North America (Baja California to Oregon), with disjunct populations occurring in Arizona. The other four hummingbird taxa (*M*. *eastwoodiae*, *M*. *rupestris*, *M*. *verbenaceus*, *M*. *nelsonii*) are each restricted to much smaller "sky-island" ranges in the southwestern U.S. and Mexico [21, 22, 24]. The bumblebee-pollinated high-elevation specialist *M*. *lewisii* is also widespread, with a dark-pink flowered Northern race found in the Rocky and Cascade Mountain ranges retained as *E. lewisii* in [24] and a pale-pink flowered Sierran race broadly parapatric with *M*. *cardinalis* in the Sierra Nevada Mountains of California renamed *E. erubescens* in [24]. Both the hummingbird- and bee-pollinated taxa are primarily perennial, occurring in soils that remain wet throughout the summer growing season. The eighth taxon, *M*. *parishii*, is a routinely self-pollinating small-flowered annual occurring in seasonally wet habitats in southern California (e.g. desert washes). Despite their distinct pollination syndromes, all these taxa are at least partially cross-compatible [25, 26] and natural hybrids have been reported between *M*. *cardinalis* and the two taxa with which it co-occurs in California (*M*. *lewisii* and *M*. *parishii*) [27]. The combination of diversity and genetic tractability has made the *Erythanthe* radiation a model for understanding the genetic basis of both floral trait divergence and species barriers for over half a century [25].

In ecological genetic work prior to the establishment of molecular phylogenetics, the extensive range overlap and relatively high cross-compatibility of Sierran *M*. *lewisii* and *M*. *cardinalis* established them as sister taxa locally adapted to distinct elevational and pollinator niches [25, 26, 28–30]. Early QTL mapping studies of species differences and barriers identified the few major loci underlying each aspect of their pollination syndromes, including nectar volume and corolla traits [28, 29], and demonstrated that these conferred pollinator specificity and assortative mating between experimental hybrids in sympatry [27, 30, 31]. It has since become clear that inferring the genetic architecture of adaptation in this pair is complicated by multiple inversions and translocations that suppress free recombination in hybrids [32, 33] and also cause underdominant F_1_ sterility [34]. However, the inference that major Mendelian genes define and isolate florally-distinct sister monkeyflowers has been strengthened by the molecular dissection of loci underlying pigmentation variants [35, 36], contributing to establishment of this group a model system for floral evolution and development (reviewed in [37]).

Sister status for parapatric *M*. *cardinalis* and *M*. *lewisii*, and the companion inference of two distinct evolutionary transitions from bee to hummingbird pollination (one in the four sky-island taxa, one more recently in *M*. *cardinalis*; Fig 1) have remained well-accepted in the post-phylogenetic era. Indeed, after phylogenetic work redefining *Mimulus* [38], re-organizing the North American sections of the genus [39] and re-tracing the evolution of hummingbird pollination in section *Erythranthe* [22], the system became a textbook example of rapid convergent evolution, as well as speciation by large-effect adaptive alleles, e.g.[40]. However, due to low resolution in universal loci used for plant phylogenetics at the time [39], the within-*Erythranthe* tree was primarily based on genome-wide population genetic markers (amplified fragment length polymorphisms, AFLPs) [22]. There are many reasons why either a few slowly-evolving loci or an aggregate of AFLPs might not clearly reflect the true evolutionary history of a given set of species, especially in a recent radiation [14]. Furthermore, while the hummingbird pollination syndrome is one of the most distinct, repeatable, and reproductively-isolating peaks in the adaptive landscape of flowering plants [41–45], inference about the genetic mechanisms of convergence and divergence in pollination syndrome among close relatives requires a well-resolved phylogenetic context. Thus, phylogenomic re-assessment of this group is an essential foundation for the study of micro- and macro-evolutionary processes in this classic system, as well as a window into the complex evolutionary histories possible in even a small radiation.

## Results and discussion

### Whole-genome species trees suggest a single origin of hummingbird pollination

We used Illumina sequencing of targeted genic regions (gene-capture; see Materials and Methods) to survey genome-wide variation within and among species in *Mimulus* section *Erythranthe*. The capture probes targeted genes 1:1 orthologous among *M*. *lewisii (v 1*.*1;* [22]*)*, *M*. *cardinalis (v 1*.*1;* *www*.*mimubase*.*org**)*, and the yellow monkeyflower *M*. *guttatus (*v2 reference; https://phytozome.jgi.doe.gov). We sequenced accessions of *M*. *lewisii* (n total = 19; per population: range = 1–4, median = 2), *M*. *cardinalis* (n total = 34; per population: range = 1–5, median = 3), and *M*. *parishii* (n = 2) from across their geographic ranges, as well as a single accession each of *M*. *verbenaceus*, *M*. *rupestris*, and *M*. *eastwoodiae* (S1 Table). Across 8,151 sequenced capture regions (7,078,270 bp total) aligned to chromosomes of the v 1.9 *M*. *cardinalis* reference genome assembly (www.mimulubase.org), we obtained 533,649 single nucleotide variants (SNVs). The bee-pollinated annual *Mimulus bicolor* was used as a close outgroup to section *Erythranthe* [22]. Whole-genome pooled population sequencing of *M*. *bicolor* revealed an additional 207,238 SNVs between *M*. *bicolor* and section *Erythranthe* within regions defined by the targeted capture sequencing, totaling 740,887 variant sites. This set of SNVs was divided across 8,151 capture regions with at least one informative site (median: 67 variable sites; IQR: 42–100; max: 316) and fully spans the physical and genetic landscape of *Mimulus* section *Erythranthe* chromosomes, thus providing a well-resolved picture of their evolutionary history.

**Fig 2:**
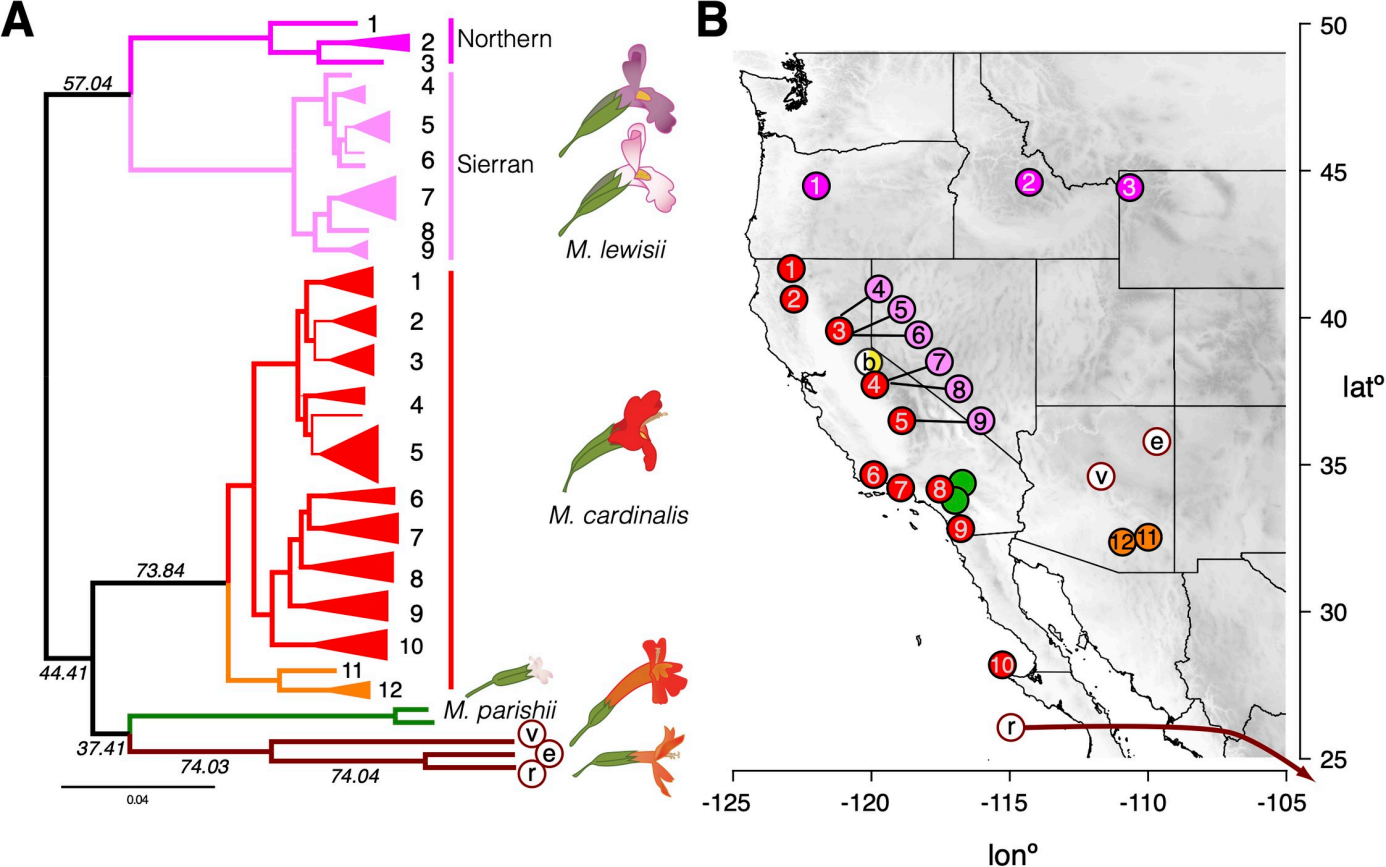
Genome-wide phylogeny of *Mimulus* section *Erythranthe* reveals a single clade containing all hummingbird-pollinated species. (A) The maximum likelihood phylogeny of section *Erythranthe* rooted to *M*. *bicolor*. The species level topology is identical to that inferred with ASTRAL 3. Branches with bootstrap support >90% are bold. Quartet scores are also given for branches included in the ASTRAL species tree. Clades representing a single collection location are collapsed (see S18 Fig for the unrooted phylogeny including the branch to *M*. *bicolor*). Numbers next to *M*. *lewisii* and *M*. *cardinalis* tips refer to collection locations in B. (B) Collections of section *Erythranthe* across the American West. Sierran *M*. *lewisii* collections are offset due to close overlap with *M*. *cardinalis* collections in the Sierra Nevada Range. Location of the *M*. *rupestris* accession from Central Mexico not shown (see S1 Table). ‘b’: *M*. *bicolor*; v: *M*. *verbenaceus*; ‘e’: *M*. *eastwoodiae*; ‘r’: *M*. *rupestris*.

We inferred phylogenetic relationships among species in Section *Erythranthe* using maximum likelihood inference of the full dataset using IQ-TREE [46] and TreeMix [47] and by assessing variation in gene tree topologies under the multispecies coalescent (MSC) with the software ASTRAL III [48]. All methods produced identical species relationships (Figs 2, S1 and S2). All species-level branches had 100% bootstrap support (IQ-TREE) and local posterior probabilities of 1 (ASTRAL). ASTRAL quartet scores (i.e. the proportion of underlying gene trees that support a branch in the species tree) ranged from 37.4 to 74.0. Branches closer to our inferred root tended to have lower quartet scores, meaning that a smaller proportion of individual gene trees supported these branches. We interpret the high level of discordance between the species tree and individual gene trees on highly supported branches as the combined effect of ILS and introgression (see below) during the early divergence of ancestral populations.

Phylogenetic and phylogeographic patterns within and between *Mimulus lewisii* and *M*. *cardinalis* are particularly important, given their status as a model system for understanding speciation. Each species formed a monophyletic clade with 100% bootstrap support and phylogeny strongly reflected geography within each. We find a deep split between *M*. *lewisii* from the Sierra Nevada Range in California (Sierran *M*. *lewisii; E*. *erubescens*) and *M*. *lewisii* from the northern Cascade Range and Rocky Mountains (Northern *M*. *lewisii; E*. *lewisii*). This result supports the long-held designation of these two clades as ‘races’ [25] or species [24] (hereafter ‘clades’), based on disjunct ranges, distinct vegetative and floral characters, and partial incompatibility and sterility in some hybrid crosses. *M*. *cardinalis* was also structured geographically, with accessions from Arizona, named *E. cinnabarina* in [24] forming an outgroup to *M*. *cardinalis* from the Pacific coast. Within the Pacific clade, *M*. *cardinalis* from southern California and northern Baja California were monophyletic and sister to a clade containing *M*. *cardinalis* from the Sierra Nevada. Consistent with the trees, genetic diversity within *M*. *lewisii* was heavily structured between Northern and Sierran *M*. *lewisii* (median *d*_*XY*_: 0.0117, IQR: 0.0074–0.0170), and Northern *M*. *lewisii* was substantially more diverse (median π: 0.0037; IQR: 0.0016–0.0071) than *M*. *lewisii* in the Sierra Nevada Range (median π: 0.0015; IQR: 0.0006–0.0042). *M*. *cardinalis* had levels of nucleotide diversity (median π: 0.0036; IQR: 0.0021–0.0060) similar to Northern *M*. *lewisii* and was more divergent from Sierran *M*. *lewisii* (median *d*_*XY*_: 0.0151, IQR: 0.0103–0.0203) than the populations of *M*. *lewisii* were from each other. Observed heterozygosity in *M*. *cardinalis* decreased with latitude, supporting the hypothesis that the current range of *M*. *cardinalis* is the result of a recent northward expansion [49]. Additional work will be necessary to determine whether the geographical isolates of both *M*. *lewisii* and *M*. *cardinalis* represent fully-fledged species. Regardless, these phenotypically subtle geographic clades make *Erythranthe* an interesting model system for understanding the evolution of postzygotic barriers in allopatry, as well as for the radiation of traits involved in pre-mating isolation in sympatry.

Despite within-species consistency with the previous section *Erythranthe* phylogeny [22], our species tree differs radically in the placement of *M*. *cardinalis* and *M*. *parishii*: both are included in a single clade which also contains all other hummingbird-pollinated species (hereafter referred to as Clade H) (Fig 2). The implications for this revision are three-fold. First, the early history of section *Erythranthe* is primarily defined by the split between the ancestor of *M*. *lewisii* and the common ancestor of all other species in the group. Second, the model pair of *M*. *lewisii* and *M*. *cardinalis* do not share recent common ancestry, at least not to the exclusion of any other species in the section. Third, the placement of all red-flowered species in a single clade strongly suggests that the hummingbird pollination syndrome evolved only once in this group and thus is not a case of phenotypic convergence. We therefore address three further questions raised by this inference and its contrast to previous work. Do key hummingbird-associated floral traits in *M*. *cardinalis* and other red-flowered species share a functional basis? What is the genomic evidence for and against close evolutionary relationships between *M*. *cardinalis*, *M*. *lewisii*, and *M*. *parishii*? What evolutionary processes are responsible for cross-genome heterogeneity of gene trees in this recent radiation?

### Key floral traits in red-flowered species appear to share a functional genetic basis, also consistent with a single evolutionary origin of hummingbird pollination

To further investigate whether *M*. *cardinalis* and the sky-island endemics plausibly share a functional basis for floral traits associated with hummingbird pollination, we conducted a classic genetic complementation test (see Material and Methods). Key hummingbird syndrome traits of both *M*. *cardinalis* [28, 29, 32] and the sky-island taxa (e.g. *M*. *rupestris*) are largely recessive to *M*. *lewisii* (as well as *M*. *parishii*), with F_1_ hybrids between bee- and hummingbird-pollinated taxa remarkably *M*. *lewisii*-like in most floral traits (S3A Fig). Under the historical scenario of convergent evolution from an ancestor resembling bee-pollinated *M*. *lewisii*, any alleles conferring the hummingbird-associated trait (e.g., highly exserted stigmas, narrow corolla apertures, high production of nectar and carotenoid and anthocyanin pigments) would be independent mutations fixed in each lineage. Thus, unless each series of (at least partially recessive) mutations non-functionalized the same set of target genes, we would expect transgressive variation in F_1_ hybrids between the putatively convergent hummingbird taxa. For the completly recessive pigment traits: if a causal *a* allele for carotenoid production in *M*. *cardinalis* (*aaBB*) is not allelic (functionally interchangeable or identical by descent) with the independent *b* allele underlying the phenotype in another taxon (e.g., *M*. *rupestris* or *M*. *verbenaceus; AAbb*) the recessive carotenoid phenotype should be masked in F_1_ hybrids (*AaBb*). We see precisely the opposite—the flowers of F_1_ hybrids between *M*. *cardinalis* and *M*. *rupestris* or *M*. *verbenaceus* resemble the parents in all respects, with no transgressive *M*. *lewisii*-like variation (S3B and S3C Fig). Substantial hybrid breakdown leading to sterility and floral deformation leads to segregation beyond parental and F_1_ values in F_2_s, but there is no evidence of hybrids reverting to the dominant *M*. *lewisii*-like phenotype expected if the genetic basis for the syndrome is not shared (see Materials and Methods). Redundant loss-of-function mutations or epistatic interactions in highly constrained pigmentation pathways could possibly produce these patterns for corolla color [42]; however, the complementation of the overall floral morphology is best explained by at least partial allelism of the mutations underlying shared aspects of the hummingbird pollination syndrome. More work will be necessary to understand the molecular and evolutionary genetics of floral divergence across section *Erythranthe*. However, this genetic evidence of non-independence corroborates the phylogenetic inference that hummingbird pollination evolved in a common ancestor of *M*. *cardinalis* and the sky-island endemics, challenging a classic case of convergence and providing a new framework for understanding adaptation and speciation in this model group.

Together, our genomic and experimental results underline the necessity of an explicitly phylogenomic context for understanding trait evolution and speciation in rapid radiations. Hummingbird pollination undoubtedly evolves convergently both within [50] and among [2, 41, 51] genera, but pollination syndromes may be particularly prone to complex evolutionary histories that mimic phenotypic convergence at low phylogenetic resolution. Like anti-predator mimicry phenotypes in *Heliconius* butterflies [11], specialized pollination syndromes (e.g., hummingbird, moth) evolve to match a pre-existing model [52]. This creates alternative multi-dimensional adaptive peaks separated by valleys of low fitness, although self-pollination may flatten this landscape [53]. Thus, the path from bee to hummingbird pollination appears to be a very narrow and sequential one–that is, a red-flowered mutant without the expected nectar reward or reproductive parts long enough for effective hummingbird pollination may be a poor match for any pollinator [30, 54]. Importantly, a jagged adaptive landscape for pollination syndromes may also mean that the joint introgression of multiple traits or their joint retention in the face of homogenizing gene flow (as inferred here) may be common whenever gene exchange occurs during floral diversification. Both processes may mimic true convergence at a coarse phylogenetic scale, but more resemble the repeated re-use of ancient alleles during freshwater adaptation in stickleback populations [55]. As phylogenomic approaches increasingly allow gene-scale investigation of deeper radiations, and more adaptive genes are identified, such sharing of old variation may often be revealed to underlie trait diversification and parallelism, even in otherwise well-resolved species [18, 56].

Given the revision of the species tree, it is also worth revisiting the inference that bee-pollination is ancestral [22], especially given the presence of yellow carotenoid pigments in both outgroup taxa such as (bee-pollinated) *M*. *bicolor* and the hummingbird-pollinated *Erythranthe*. Across flowering plants, transitions from bee to hummingbird pollination appear far more likely than the reverse [45], due either to genetic constraints [50] and/or the ecology of pollination [54]. Bees tend to ignore red flowers and have nowhere to land on narrowly tubular and reflexed “hummingbird” corollas whereas hummingbirds often visit classic bumblebee flowers; for example, hummingbirds made nearly 20% of the visits to Sierran *M*. *lewisii* in experimental arrays with *M*. *cardinalis* and hybrids [31]. Even a low frequency of “mistakes”, especially when hummingbird visits are abundant and bees rare, may select for hummingbird-specialization through increased reward, greater attraction, and more precise pollen placement. In this system, where the bee-specialized pale pink flowers and scent production of Sierran *M*. *lewisii* (*E*. *erubecens*) appear locally derived [36, 57, 58], it is plausible that hummingbird visitation to a less-specialized Northern *M*. *lewisii*-like ancestor precipitating the origin of hummingbird pollination within Clade H. However, ancestral hummingbird pollination remains formally possible and confirming the expected directionality will require reconstruction of the mutational changes contributing to key trait transitions across the entire radiation.

### Extensive introgression creates the evidence for a sister relationship between *M*. *lewisii* and *M*. *cardinalis*

**Fig 3:**
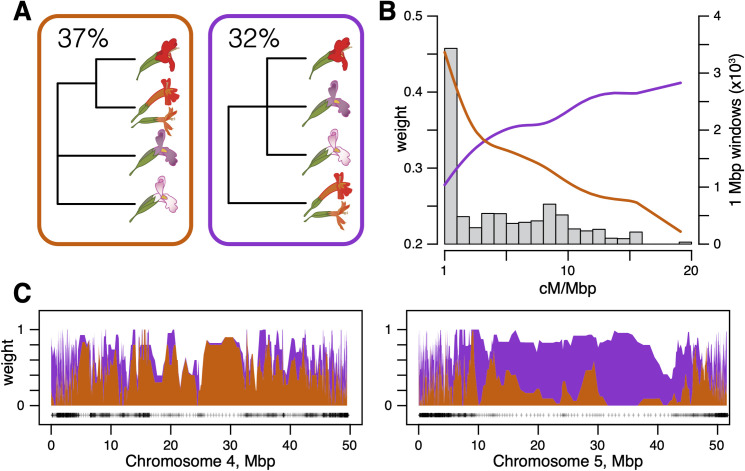
Introgression has generated the evidence for sisterhood of *M*. *lewisii* and *M*. *cardinalis*. (A) Genome-wide TWISST weightings for a simplified species tree (orange) and a simplified “lew-card” tree (purple). (B) Support for the species and lew-card trees as a function of recombination rate. Lines show cubic spline fits colored as in A. The gray histogram shows the frequency of genomic windows at a given recombination rate (bin size: 1 cM/Mbp). (C) Topology weights along *M*. *cardinalis* Chromosomes 4 and 5. Polygons are stacked so that weights across all possible topologies (including those not shown) sum to 1. Weights are averaged in windows of 5 genes; black crosses show locations of window midpoints. See S7–S14 Figs for topology weights across all 8 *M*. *cardinalis* chromosomes.

Because they are a decades-old model system for understanding the role of reproductive adaptation in plant speciation, general inferences about the nature of those processes hinge on *M*. *cardinalis* and *M*. *lewisii* being parapatric sister species. Moreover, the initial inference of a close relationship was plausibly based on similar vegetative morphology, shared geography, and higher genetic compatibility between the Sierran pair than geographically-disjunct populations within each species [25], as well as previous phylogenetic reconstructions [22]. Given that our whole-genome species tree robustly rejects close sister status for *M*. *lewisii* and *M*. *cardinalis*, placing *M*. *cardinalis* within the predominantly hummingbird-pollinated Clade H, it is important to understand the origins of these confounding affinities. Therefore, we examine our genomic dataset for evidence of a close relationship, describe the genomic distribution of regions showing a sister relationship, and infer the processes underlying patterns of gene tree vs. species tree discordance. We used TWISST [59], which quantifies support for different species tree topologies among a set of inferred gene trees, to compare support for trees containing Clade H (all red-flowered species, the ‘species tree’; Fig 3A, orange) to support for trees where *M*. *lewisii* and *M*. *cardinalis* form an exclusive clade (the ‘lew-card tree’; Fig 3A, purple). TWISST samples subtrees (subsets of each gene tree that contain a single sampled tip of each species of interest) across all gene trees and uses their frequencies within and among gene trees as evidence of alternative evolutionary histories. Because we were primarily interested in the relationships between these two focal species, we were agnostic to the placement of *M*. *parishii* in these analyses. Notably, the lew-card tree was the second-most common topology observed across the genome, next only to our inferred species tree (Fig 3A). Across the entire dataset consisting of 8,151 gene trees, 37% of subtrees identified in TWISST supported the species tree while 32% supported the ‘lew-card’ tree. Substantial incomplete lineage sorting (ILS) at the base of this radiation could produce this pattern, but we hypothesized that introgression between *M*. *lewisii* and *M*. *cardinalis* was a more likely source given current parapatry and cross-compatibility. Therefore, to explore introgression as source of gene-tree/species-tree discordance, we tested for (1) asymmetries in patterns of shared, discordant allelic states among species, (2) patterns of absolute genetic divergence indicative of a reticulate evolutionary history, and (3) a correlation between recombination rate and support for the ‘lew-card’ tree.

We first tested for genome-wide evidence of the presence, timing, and direction of introgression between *M*. *lewisii* and *M*. *cardinalis* using Patterson’s D statistic [60] and D_FOIL_, a five-taxon expansion of Patterson’s D [61]. Patterson’s D–also known as the ABBA-BABA test–identifies introgression based on shared allelic states among species on a four-taxon tree: variant sites that do not support the species tree should support alternative trees at equal frequency under incomplete lineage sorting, while introgression will upset this balance. We detected significant introgression between *M*. *cardinalis* and *M*. *lewisii* (block jackknife: z-score = 3150.844; *p* < 2 x 10^−308^). The magnitude of D depended on which accessions of *M*. *cardinalis* and *M*. *lewisii* were used in the test (range: 0.01–0.10), but D was always non-zero (S4 Fig), indicating that introgression was not restricted to a single portion of the current species ranges. Bolstering and refining this inference, the predominant introgression signal detected by D_FOIL_ was between *M*. *cardinalis* and ancestral *M*. *lewisii* (i.e., prior to divergence of its Sierran and Northern clades) (S5A and S5B Fig).

The early timing of inferred introgression prevented assessment of its direction with D_FOIL_ alone [61]. We then used an additional test, D2 [62], which infers the direction of introgression using expectations from the multispecies network coalescent. Directional introgression from *M*. *cardinalis* into *M*. *lewisii* would result in reduced nucleotide divergence between *M*. *lewisii* and the other species of Clade H (e.g. *M*. *verbenaceus*) at genes following the introgression tree (S5C and S5D Fig). This is because these alleles sampled from *M*. *lewisii* are historically *M*. *cardinalis* alleles and reflect divergence between *M*. *cardinalis* and the rest of Clade H. In contrast, introgression from *M*. *lewisii* into *M*. *cardinalis* would not affect sequence divergence between *M*. *lewisii* and non-*cardinalis* members of Clade H. We detected no difference in sequence divergence between *M*. *lewisii* and third taxon *M*. *verbenaceus* at genes whose history matched the species tree versus the introgression tree (t-test: *t*_3354.3_ = 1.12, p = 0.26; S5D Fig). Therefore, we infer that introgression during this early period mostly moved genetic material asymmetrically from ancestral *M*. *lewisii* into *M*. *cardinalis*.

In addition to producing asymmetric allele-sharing on a phylogeny, the distribution of introgressed DNA should vary predictably across the genome. In particular, the extent to which neutral introgressed variation establishes or fixes in a recipient population should be strongly affected by the local recombination rate (reviewed in [15]). At one extreme, adaptive (or selfish) introgression of a mitochondrial sequence variant could carry both the entire mitochondrial genome and linked chloroplast variants to fixation across species boundaries [63]. However, the more plausible assumption is that the vast majority of genomic segments carry variants that are either neutral or deleterious in a heterospecific background. Because low recombination rates extend the effects of selection against deleterious incoming alleles over larger physical regions, such regions may be broadly protected from introgression. In contrast, variants in high-recombination regions are affected by selection on their individual merits, allowing rates of (neutral or beneficial) introgression to be higher.

To investigate the relationship between recombination rate and introgression in the *Erythranthe* group, we used a dense linkage map of *M*. *cardinalis* generated from a subset of gene-capture loci [64]. This map supported a chromosome-level scaffolding of *M*. *cardinalis and M*. *lewisii* genomes (since reinforced with additional data to form the current V2 genomes; www.mimubase.org) and allows confident genetic-physical comparisons (see Materials and Methods). Crossovers in *M*. *cardinalis* occur almost exclusively on the ends of each chromosome, with very little recombination across large, presumably centromeric and pericentromeric, central regions (S6 Fig**).** The species tree was the most common topology observed in these low- or non- recombining regions, which also covered ~68% the physical expanse of the genome (i.e. contigs scaffolded with the genetic map; 235/345 1Mbp windows; Figs 3B and S7). Support for the lew-card tree was strongly and positively correlated with recombination rate (Spearman’s ρ = 0.136, p = 1x10^-10^; Fig 3B), with the introgression topology becoming predominant at recombination rates > 2.5 cM/Mbp. Indeed, this pattern is so pervasive that maximum likelihood phylogenies place *M*. *cardinalis* as sister to (IQ-TREE) or nested within (TreeMix) *M*. *lewisii* when we limit our dataset to freely recombining sites. When we inferred the maximum likelihood phylogeny with IQ-TREE using only SNVs in windows with recombination rates greater than 5 cM/Mbp, *M*. *lewisii* and *M*. *cardinalis* came out as sister taxa with 100% bootstrap support (S15 Fig). Using TreeMix with an LD-pruned dataset (pairwise r^2^ ≤ 0.50 for included SNVs) to better match the assumptions of the model [47] placed *M*. *cardinalis* sister to Sierran *M*. *lewisii* (S2D Fig).

Although elevated introgression only at chromosome ends was the dominant genome-wide pattern, we also observed near-complete replacement of some chromosomes that erased the underlying species tree (Figs 3C and S7–S14). For example, Chromosome 5 consistently supports the ‘lew-card’ tree, including across its low-recombination central region (Fig 3C). In contrast, Chromosome 4 generally showed high support for the species tree (Fig 3C). Chromosome 4 contains multiple ecologically-relevant quantitative trait loci (QTLs) in crosses between *M*. *lewisii* and *M*. *cardinalis*, including the ‘yellow upper’ (YUP) locus [28], which switches petal color from pink/purple to red via carotenoid deposition. YUP is embedded in a large region of completely suppressed recombination in *M*. *lewisii* x *M*. *cardinalis* mapping crosses (likely an inversion), and is in tight linkage with a major flower length QTL and a putative hybrid lethality factor [32]. Strong selection against heterospecific alleles and low recombination in hybrids may make this entire chromosome particularly resistant to introgression in areas of ancestral or recent contact between *M*. *lewisii* and *M*. *cardinalis*.

Our results corroborate one of most striking results of speciation genomics over the past decade: introgression between closely related species is widespread and can profoundly affect the course of evolution. The extent of introgression ranges from one or a few loci involved in adaptation [11, 65] to genome-wide exchange that nearly swamps out past population histories [66–68]. Our phylogenomic results place introgression between *M*. *lewisii* and *M*. *cardinalis* near the upper end of this continuum, so it is not surprising that past sampling of loci could infer other histories [22]. Similar patterns have been seen in *Anopheles* mosquitoes [67] and among some cat species [68], where the predominant genome-wide signal derives from hybridization. In those animal cases, strong hybrid F_1_ incompatibilities map to the sex chromosomes, giving them extra weight in inferring the likely species tree. Here, we resolve speciation histories only because these *Mimulus* genomes contain large pericentromeric regions that rarely recombine and are generally resistant to gene exchange. The resulting species-tree inference is bolstered by a strong chromosome-scale match from a key adaptive chromosome (Chromosome 4) underlying multiple pollination-syndrome traits. Within the physically small, but highly recombining and gene-dense ends of chromosomes, admixture predominates. The latter pattern strongly supports our inference that introgression, rather than a recent split, creates signals of sisterhood between *M*. *lewisii* and *M*. *cardinalis*.

### Despite strong reproductive barriers between *M*. *cardinalis* and *M*. *lewisii*, recent introgression (including chloroplast capture) has occurred in their shared Sierran range

Although broadly parapatric, Sierran *Mimulus lewisii* and *M*. *cardinalis* are reproductively isolated from one another by a series of strong but incomplete barriers [25, 27]. Ecogeographic isolation [27], elevational specialization [69] and distinct pollination syndromes [30] result in near-complete pre-mating isolation. In addition, a pair of intrinsically underdominant chromosomal translocations make F_1_ hybrids >65% pollen-sterile [32, 34]. Despite these strong contemporary barriers, we also find substantial evidence of recent introgression (both nuclear and organellar) where *M*. *lewisii* and *M*. *cardinalis* co-occur in the Sierra Nevada Range of California. Sierran *M*. *lewisii* and *M*. *cardinalis* formed a monophyletic clade in 14.5% of nuclear subtrees analyzed with TWISST; this clade was fully supported at 5.9% of gene trees (479 of 8151). These percentages are generally a subset of those trees in Fig 3A that support a *M*. *lewisii-M*. *cardinalis* clade but do not specify a branching pattern within that clade. This more recent introgression event also corresponds to the predominant migration edge inferred with TreeMix (S2 Fig). TreeMix analysis also inferred that the direction of recent introgression was from *M*. *cardinalis* into *M*. *lewisii*.

**Fig 4:**
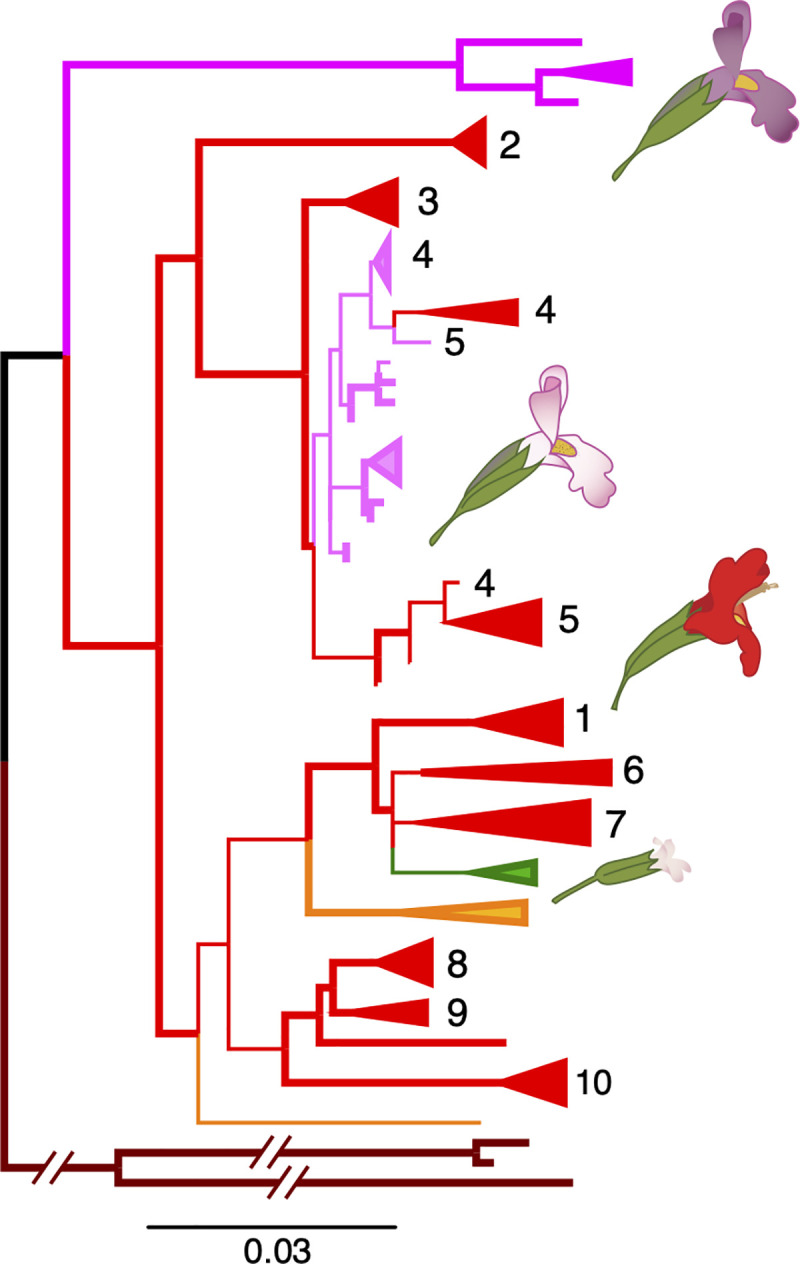
The chloroplast phylogeny demonstrates ancient and recent, geographically local, introgression. The maximum likelihood phylogeny rooted to *M*. *bicolor* is shown. Long branches to *M*. *verbenaceus*, *M*. *rupestris*, and *M*. *eastwoodiae* are abbreviated (See S17 Fig for the unrooted, unabbreviated tree). Species and are colored and populations are numbered as in Fig 2. Branches with >90% bootstrap support are in bold.

Further support for directional introgression into *M*. *lewisii* comes from organellar genomes. Chloroplast haplotypes (genotyped using organellar reads skimmed from the nuclear capture data; see Materials and Methods) from Sierran *M*. *lewisii* and nearby *M*. *cardinalis* populations form a single clade (100% bootstrap support; Figs 4 and S17). Due to short branch-lengths, we conservatively consider the base of the Sierran *M*. *lewisii* clade to be a polytomy; however, moderate bootstrap support (62%) for monophyly of the *M*. *lewisii* haplotypes suggests that a single local *M*. *cardinalis* cytoplasm may have recently swept through all Sierran *M*. *lewisii* populations. Importantly, the shared Sierran range where we infer organellar transfer is the source for the accessions of both species used in previous adaptation and speciation genetic studies, phylogenetics [39], and reference genome assemblies.

More work will be necessary to understand whether organellar (and nuclear) introgression in the Sierras represents “surfing” of neutral variation introduced from an expanding *M*. *cardinalis* range-front [70] or the spread of adaptive or selfish alleles by natural selection. In either case, strong evidence of recent organellar capture [19] reinforces the inference of ancient and recent nuclear introgression in this system, and further suggests that strong ecological and genetic barriers have not been sufficient to isolate the entire genomes of these young taxa upon secondary contact. Although natural hybridization between *M*. *lewisii* and *M*. *cardinalis* is rare [27] and costly [27, 34], a little gene flow goes a long way [71]. This evidence for recent (as well as ancient) introgression re-iterates the importance of an evolutionary genomic framework for understanding the process of speciation, and also underlines the potential for hybridization (even between highly isolated taxa) as a source of beneficial alleles for contemporary evolution in response to changing environments.

### Organellar capture by selfer *M*. *parishii* confirms local hybridization with *M*. *cardinalis*, and may explain cytoplasmic male sterility in its hybrids with *M*. *lewisii*

In a second case of recent introgression, the chloroplast tree shows that selfing species *M*. *parishii* has captured the cytoplasmic genomes of the outcrossing *M*. *cardinalis* (Fig 4). Specifically, *M*. *parishii* chloroplast haplotypes are nested within *M*. *cardinalis* variation from their region of range overlap in Southern California. As with the transfer of local *M*. *cardinalis* organelles into Sierran *M*. *lewisii*, this geographical signal strongly supports recent introgression over alternative sources of phylogenetic discordance. Despite *M*. *parishii*’s floral adaptations for self-pollination (tiny pale-pink flowers with little nectar and no separation of male and female organs; Figs 1 and S3A), hybrids between the selfer and *M*. *cardinalis* have been reported where they co-occur along ephemeral waterways. Given the difference in mating system, we might expect that F_1_ hybrids would have selfer seed parents and would backcross primarily to the outcrossing species, causing introgression of nuclear genes from *M*. *parishii* into *M*. *cardinalis*, as seen in the yellow monkeyflower pair, *M*. *nasutus* (selfer) and *M*. *guttatus* (outcrosser) [72, 73]. Instead, the highly selfing species appears to have captured the organellar genome of the outcrossing species. This may have been made more likely by the general dominance of *M*. *parishii* for floral traits (S3A Fig); in a hybrid swarm, selfing (rather than backcrossing to the outcrossing taxon) may be the primary mode of pollination.

Recent introgression between these highly divergent taxa may also help explain the puzzling cytoplasmic male sterility (CMS; anthers produce no pollen) in hybrids between *M*. *parishii* and *M*. *lewisii* [33]. In that study, we found that F_2_ hybrids with the *M*. *parishii* cytoplasm exhibit CMS if they do not also carry *M*. *parishii* alleles at multiple nuclear restorer loci, whereas reciprocal hybrids do not exhibit anther sterility. CMS in flowering plant hybrids is common and thought to result from selfish male-sterilizing mitochondrial haplotypes [74] that spread within species by slightly increasing female fitness, in turn favoring the spread of matched nuclear restorers of male fertility [75]. Selfish CMS-restorer dynamics are theoretically plausible and have been empirically demonstrated in other *Mimulus* species [76], but should not occur in highly selfing taxa where individual female fitness also depends on some pollen production [77]. However, conditions for the spread and establishment of an heterospecific CMS variant, which can co-introgress with its (dominant) restorer allele, may be less restrictive than on a *de novo* CMS mutation. Thus, while *M*. *parishii* x *M*. *lewisii* CMS could still reflect independent neutral divergence at the hybrid-interacting loci, *M*. *parishii*’s possession of an organellar haplotype recently transferred from neighboring *M*. *cardinalis* revives the possibility of a selfish history for this asymmetric hybrid incompatibility.

## Conclusions

**Fig 5:**
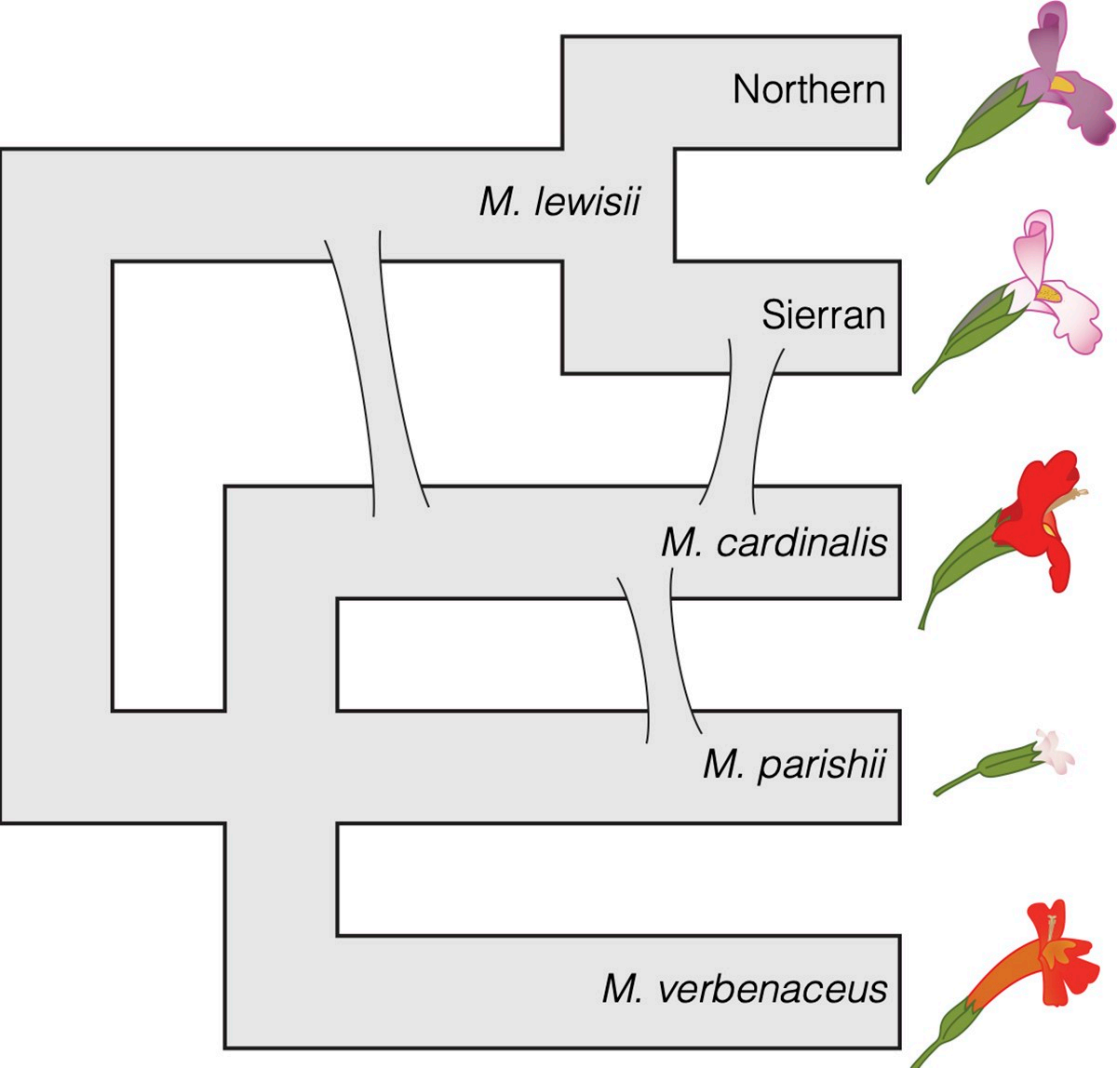
A revised evolutionary history of *Mimulus* section *Erythranthe*. The three major introgression events shown contribute to discordance between previous molecular phylogenies and the revised species tree. The ‘slope’ of each reticulation indicates the inferred direction of introgression, where the upstream species is the donor and the downstream species the recipient of introgressed alleles. Clade H is shown as a tritomy due to long external branches and short internal branches; however, it is plausible that *M*. *parishii* and a hummingbird-pollinated ancestor of *M*. *cardinalis* and *M*. *verbenaceus* were both separately derived from an (large, potentially structured) ancestral population that phenotypically resembled Northern *M*. *lewisii*.

Our understanding of adaption and speciation is contingent on understanding the demographic and genetic histories of diverging populations, which the genomics era is proving to be remarkably reticulate. We present the first population genomic dataset in the classic model system of *Mimulus* section *Erythranthe* to clarify the history of species divergence and reveal rampant introgression during periods of secondary contact. Definitive work on patterns of reproductive isolation [25, 27], abiotic [69] and biotic [30]adaptation, convergence in pollination syndromes [22]and speciation genetics [28, 34] have been built on the foundation of close sister status for sympatric *M*. *lewisii* and *M*. *cardinalis*. However, these model taxa join a growing number of systems in which introgression shapes trait evolution relevant to speciation and obscures deeper histories of divergence. Our analyses suggest that introgressive hybridization–and not recent parapatric speciation–is primarily responsible for the signals of genetic closeness captured in previous phylogenetic analyses (Fig 5). Gene flow between *M*. *lewisii* and *M*. *cardinalis*, both in the past and in their current zone of sympatry in the Sierran Nevada Range, causes much of the nuclear genome to support sister species status. Multiple instances of geographically restricted cytoplasmic introgression reinforce the inference of pervasive hybridization in this system and may also explain the paradoxical cytoplasmic male sterility (CMS) of selfer *M*. *parishii*. Importantly, our revision of the species tree for *Mimulus* section *Erythranthe* demonstrates that long-term resistance to introgression, rather than convergence, may be important in shaping multi-trait pollination syndromes during adaptive radiation in complex landscapes. While shifting the genetic origin of the hummingbird pollination system to an earlier branch, our genome-wide evidence for reticulation during the *Erythranthe* radiation only enriches its value for understanding the origins and maintenance of species barriers. The layers of pre- and post-zygotic isolating mechanisms in current contact zones built up over time and space, thus providing the opportunity to excavate their evolution and interactions across the entire radiation.

## Materials and methods

### Collections and plant material

We obtained wild-collected seeds from throughout the geographic range of *Mimulus* section *Erythranthe* with particular focus on *M*. *lewisii* and *M*. *cardinalis* populations (Fig 2 and S1 Table). Plants were grown from seed in a greenhouse at the University of Montana and DNA extracted from leaf or flower bud tissue using a customized CTAB-chloroform extraction protocol (dx.doi.org/10.17504/protocols.io.bgv6jw9e). We used *M*. *bicolor* as an outgroup species to the core *Erythranthe* taxa, as has been done previously; however, due to loss of *M*. *bicolor* DNA from the capture-sequencing set, we used PoolSeq data from a separate study to obtain high-coverage data from target regions. Whole *M*. *bicolor* plants (n = 160) were wild-collected from a large color-polymorphic population in center of its range in the Sierra Nevada Range [78] and dried in coin envelopes, and then DNA was extracted from tissue individually prior to equal-volume pooling.

### Linkage mapping and recombination rates

We used the *M*. *cardinalis* linkage map reported in [64] and CE10 v1.92 genome contigs (www.mimubase.org) to estimate genetic and physical distances along the *M*. *cardinalis* genome. Briefly, a Sierran (CE10 inbred line) x Southern (WFM) *M*. *cardinalis* F_2_ mapping population (N = 93) was genotyped using the same targeted capture approach as this study. 8100 snps (representing 2152 cross-informative capture targets) were ordered with Lep-MAP3 [79], resolving the expected 8 linkage groups (2N = 16) spanning 573 centiMorgans (cM) [64]. The linkage map was used to scaffold v1.92 contigs with Chromonomer version 1.08 [80]. We were able to scaffold a total of 341.8 Mb of genome sequence, which is 83.6**%** of the current v2 chromosomal assembly (based on both optical mapping and linkage relationships; www.mimubase.org). The genome scaffolding used here for genome scans is largely similar in order to the v2 assembly, but its contig positions and orientation are based solely on intraspecific recombination. Recombination rates were estimated in non-overlapping genomic bins of 1 Mbp. Rates were calculated as the genetic distance (in cM) between the two most distal markers in the bin divided by their physical distance (in Mbp). We removed three bins with extreme recombination rate estimates (>100 cM/Mbp) from further analysis. These estimates were due to many crossovers between putatively physically proximal markers (<5,000 bp) with no other markers present in the bin, and likely represent mislocalization of a marker on the physical sequence (e.g., due to paralogy).

### Targeted capture sequencing and genotyping

Targeted sequence capture was used for high-coverage, high-quality genotyping within and among species in *Mimulus* section *Erythranthe*. Capture baits were designed to tile 9,126 genes that are 1:1 orthologous between *M*. *cardinalis*, *M*. *lewisii*, and *M*. *guttatus*. Details of bait design and library preparation can be found in [64]. All libraries were sequenced on a single lane of Illumina HiSeq 2500 (PE 125). Raw Illumina reads were quality filtered and trimmed for sequencing adaptors using Trimmomatic [81] and aligned to the v1.9g draft *M*. *cardinalis* genome (http://mimubase.org/FTP/Genomes/) using bwa-mem v0.7.15 [82]. Alignments were filtered for minimum quality scores of 29 using samtools v1.3 [83]. We then removed potential PCR duplicates and realigned around indels using Picard Tools (http://broadinstitute.github.io/picard) and GATK (v3.3-0-g37228af) [84] following GATK best practices.

### Pooled population sequencing of *M*. *bicolor*

*Mimulus bicolor* DNA (N = 160 wild plants from a large population) was pooled into a single sample for this study. Illumina library preparation and sequencing on an Illumina HiSeq 4000 were performed by Novogene Corporation (Stockton, CA, USA) following manufacturer protocols. Genotypes were called as above with the exception of two alterations intended to convert pooled genotypes into a single *M*. *bicolor* reference alignment. First, during GVCF creation, we instructed the GATK tool HaplotypeCaller to attempt to remove ‘contaminant’ reads at frequencies of up to 10**%** in order to remove low-frequency polymorphisms present in the pool. After VCF creation, we converted remaining heterozygous sites to homozygotes by randomly selecting one of the two alternate alleles. Multi-allelic sites were all ignored in the final analyses. Observed sequence divergence between *M*. *bicolor* and *M*. *cardinalis* (median d_xy_: 0.0277) was similar to levels of synonymous site diversity observed within a single population of the genus’s flagship species, *M*. *guttatus* [85] aligned and genotyped using the similar parameters. Additionally, observed *M*. *bicolor—M*.*cardinalis* sequence divergence was nearly identical to *M*. *bicolor—M*. *lewisii* divergence (median d_xy_: 0.0282). These results indicate that reference bias is of relatively low concern in this largely genic dataset, despite its phylogenetic scope.

### Gene tree and species tree inference

To generate a set of genomic regions representing individual protein-coding genes, we aligned capture bait sequences to the contig-level *M*. *cardinalis* v 1.9g genome assembly (http://mimubase.org/FTP/Genomes/) using BLAST v2.2.31 [86] to determine the beginning and end coordinates of each aligned bait. We then used bedtools-merge v2.26.0 [87] to merge bait alignments tiling the same gene into a single region, resulting in 8151 genomic regions. Because each capture region was designed to target a protein-coding gene, we refer to these targeted genomic regions as “genes”.

Gene tree inference and partitioned maximum likelihood (ML) phylogenetic analysis were performed on individual alignments representing each gene. We created individual alignments by extracting genotypes within the boundaries of each gene from the phased VCF using tabix [88]. Alignments thus consisted of variable sites only, and a single haplotype for each sample was included. We inferred ML phylogenies for each gene individually and the entire genome using IQ-TREE v1.7-beta14 [46] under the GTR+ASC+G4 substitution model to correct for the absence of invariant sites. This dataset included 8,151 genes in which we observed parsimony-informative sites. For the whole-genome phylogeny we also generated branch support by performing 1000 ultrafast bootstrap replicates [89]. To further ensure that the resulting phylogeny was robust to model assumptions and tree search strategies, we inferred ML trees using PhyML v20120412 [90] and RAXML v8.2.12 [91] on a concatenated super-matrix consisting of 600,267 variable sites under the GTR+gamma substitution model with four rate categories.

In addition to whole-genome concatenation, we used ASTRAL-III v5.6.3 [48] to generate a species tree under the multispecies coalescent. ASTRAL uses variation in gene tree topologies to infer a species tree under the assumption that topological discordance among gene trees is due to incomplete lineage sorting during population divergence. We ran ASTRAL on the full dataset of 8,151 gene trees inferred from IQ-TREE, using quartet scores and local posterior probabilities as branch supports. Quartet scores measure how often a given quartet (unrooted, four-taxon tree) observed in the species tree is present in the underlying gene trees. Under the assumption of no gene flow post-speciation, quartet scores are also indicative of the degree of incomplete lineage sorting along the inferred branch [92].

We used TreeMix [47] to jointly infer the maximum likelihood species topology and major migration events with allele frequency data. Species-level allele frequencies at each SNV were calculated with PLINK 1.9 [93] and converted to TreeMix input format with a custom Python script (https://github.com/thomnelson/tools/blob/master/plink2treemix.py). The model implemented in TreeMix expects input SNVs to be in approximate linkage equilibrium, so we first analyzed SNV data with stringent linkage disequilibrium pruning (plink—indep-pairwise 1000kb 1 0.50). Because phylogenetic signal in our dataset is strongly dependent on recombination and linkage, we also used TreeMix on all variable sites with minor allele frequencies ≥ 0.05 and on datasets pruned by physical distance between SNVs (100 bp and 1000 bp) (S2A–S2C Fig).

### Tree topology weighting with TWISST

We quantified variation in species relationships throughout the genome using TWISST [59]. Given a gene tree and a set of species designations for all tips in the tree, TWISST quantifies support for all possible (rooted) species trees through iterative sampling of subtrees where each species is represented by a single tip. We ran TWISST on each gene tree grouping all accessions by species except *M*. *verbenaceus*, *M*. *rupestris*, and *M*. *eastwoodiae*, which we grouped into a single ‘species.’ We did this for three reasons: (1) these species formed a single, highly supported clade in our ML and ASTRAL trees, (2) we were primarily interested in the relationships between *M*. *lewisii* and *M*. *cardinalis*, and (3) collapsing these species limited our analysis to five taxa (105 unique rooted trees) and made analysis of the entire dataset feasible (vs. seven taxa: 10,395 unique rooted trees). To quantify support among generalized species relationships (e.g. Fig 3A), topology weightings for each unique tree topology were summed across all topologies that included a clade of interest. For instance, we calculated support for the ‘species tree’ as the sum of weightings across all topologies that place *M*. *cardinalis* in a clade with the other red-flowered species. We also visualized support for different species relationships across the *M*. *cardinalis* genome by updating genome coordinates of capture regions to match the chromosome-level v2 reference assembly (www.mimubase.org). To aid in visualization, we averaged topology weights in overlapping five-gene windows.

### Genome-wide tests for introgression

We used Patterson’s D [60] and related statistics to identify aggregate genomic signatures of introgression, assuming our inferred species tree accurately reflects historical relationships within section *Erythranthe*. All tests were implemented in Python v3.5.5.

Patterson’s D statistics tested for introgression on the four-taxon tree of (*M*. *bicolor*, (*M*. *lewisii*, (*M*. *cardinalis*, *M*. *verbenaceus*))). Calculating D using *M*. *parishii* instead of *M*. *verbenaceus* produced qualitatively similar results. We used all pairwise combinations of individual accessions of *M*. *lewisii* and *M*. *cardinalis*, allowing for heterozygosity but not missing data. While D can be calculated from allele frequencies, our accessions represent multiple populations that may have experienced variable histories of introgression; pairwise calculation gave us the potential to detect geographically-limited introgression. To test for genome-wide statistical significance, we implemented the genomic window jackknife procedure suggested in [94].

D_FOIL_ statistics [61] were used to identify the timing and, potentially, the direction of introgression on the five-taxon tree (*M*. *bicolor*, ((*M*. *verbenaceus*, *M*. *cardinalis*), (Sierran *lewisii*, Northern *lewisii*))). As with Patterson’s D, we implemented D_FOIL_ in Python using individual accessions and allowing for heterozygosity but not missing data. Because the D_FOIL_ patterns we observed prevented us from inferring the direction of introgression, we calculated Hahn and Hibbins’ D2 [62]. D2 uses expectations from the network coalescent to infer the direction of introgression on a three-taxon tree. We defined the species tree as ((*M*. *verbenaceus*, *M*. *cardinalis*), *M*. *lewisii*) and the introgression tree as (*M*. *verbenaceus*, (*M*. *cardinalis*, *M*. *lewisii*)). Introgression from *M*. *cardinalis* into *M*. *lewisii* will also result in *M*. *lewisii* and *M*. *verbenaceus* sharing more recent common ancestry than at gene trees concordant with the species tree, while introgression from *M*. *lewisii* into *M*. *cardinalis* will not. We tested for this difference ([dxy_lew-verb_ | species tree]—[dxy_lew-verb_ | introgression tree]) using a t-test on genes with full TWISST weighting for either the simplified species tree or the simplified introgression tree (see Fig 3).

### Nucleotide diversity and divergence

Population genetic statistics were all calculated with the Python module scikit-allel v1.2.1

https://scikit-allel.readthedocs.io/en/stable/index.html. As input, VCF files were created that included invariant sites using the flag “—includeNonVariantSites” in the GATK tool GenotypeGVCFs. We calculated statistics on our pre-defined capture regions (‘genes’). Nucleotide diversity (π) at each gene was calculated at the species and regional levels (e.g. *M*. *lewisii* and *Sierran lewisii*) and nucleotide divergence (d_xy_) was calculated among regions and species. In the absence of a complete reference annotation for *M*. *cardinalis*, we did not differentiate among codon positions or between coding and noncoding diversity.

### Floral trait complementation test

As a rough test for allelism of genetic variation contributing to the hummingbird pollination floral syndrome of *M*. *cardinalis* and the other red-flowered taxa (specifically *M*. *verbenaceus* and *M*. *rupestris*) within the frame of the historical phylogeny, we used a classic complementation approach. First, we generated F_1_ hybrids by crossing *M*. *rupestris* and *M*. *verbenaceus* lines (S1 Table) to the putative ancestral bee-pollinated phenotype represented by *M*. *lewisii* (Sierran LF10 line) to verify that these taxa shared recessive inheritance of the hummingbird syndrome phenotype with *M*. *cardinalis*. Second, we generated F_1_ hybrids between the CE10 *M*. *cardinalis* line and *M*. *rupestris* and *M*. *verbenaceus*, and then made F_2_s by selfing a single F_1_ of each pair. We grew parents (N = 8–10), F_1_s (N = 10) and F_2_s (N = 100–200) in the greenhouse at the University of Montana. For both sets of hybrids, it was evident that key floral traits of hybrid flowers exhibited non-complementation, e.g. the long narrow-apertured corolla tube of the *M*. *rupestris* x *M*. *cardinalis* F_1_ hybrid despite complete recessivity of aperture width in wide *M*. *lewisii* x *M*. *cardinalis* hybrids [29]. Thus we measured traits on only a few plants; these quantitative measures support the qualitative inference. For example, the exsertion of the stigma beyond the corolla tube did not differ among *M*. *verbenaceus* (13.0mm ± 1.2mm SE, n = 2), *M*. *cardinalis* (15.0mm ± 0.6mm SE, n = 8), and their F_1_ hybrids (12.5 ± 0.9mm SE, n = 4), despite this trait being zero or negative (stigma inserted inside the corolla tube) in *M*. *lewisii* and at least partially M. *lewisii*-dominant (see top row of S3A Fig). However, severe hybrid breakdown (e.g., deformed corollas and styles, sterile anthers) was common in both sets of F_2_s (S3B and S3C Fig). Due to the transgressive variation introduced by floral breakdown (and because we did not grow *M*. *lewisii* at the same time for direct comparisons), we did not conduct quantitative analyses of the F_2_ floral traits.

## Supporting information

S1 Fig Unrooted ASTRAL tree demonstrating the same species-level topology as the maximum likelihood phylogeny. Lengths of internal branches are in coalescence units, as are external branches for species with >1 sample (*M*. *lewisii*, *M*. *cardinalis*, *M*. *parishii*). *M*. *cardinalis* samples are split into the Arizona clade (orange) and the California clade (red). Quartet scores for each internal branch are shown; all internal branches have local posterior probabilities of 1.
(TIF)

S2 Fig Maximum likelihood phylogenies and migration edges inferred with TreeMix. All plots use data pruned for minor allele frequencies ≥ 0.05 and tree inference with two migration edges. A. Using all available SNPs recapitulates the species relationships inferred with IQ-TREE and ASTRAL. The major migration edge corresponds to the recent introgression event from *M*. *cardinalis* into *M*. *lewisii* in the Sierra Nevada Range. B. Data pruned to exclude SNPs in close physical proximity (100 bp). C. Extended pruning to 1000 bp. D. Using LD-based pruning of SNPs with pairwise R^2^ ≥ 0.50.
(TIF)

S3 Fig Recessivity of major hummingbird-syndrome floral traits and complementation test. A. *Mimulus* section *Erythranthe* species (represented by reference inbred lines) and F_1_ hybrids. Top row: *M*. *lewisii*, *M*. *lewisii* x *M*. *cardinalis* F_1_, *M*. *lewisii* x *M*. *rupestris* F_1_. Middle row: *M*. *parishii*, *M*. *parishii* x *M*. *cardinalis* F_1_, *M*. *parishii* x *M*. *rupestris* F_1_. Bottom row: *M*. *cardinalis*, *M*. *rupestris* x *M*. *cardinalis* F_1_, *M*. *rupestris*. B. *M*. *rupestris* x *M*. *cardinalis* F_2_ hybrids. C. *M*. *verbenaceus* x *M*. *cardinalis* F_2_ hybrids.
(TIF)

S4 Fig Genome-wide Patterson’s D for all pairwise combinations of *M. lewisii* and *M. cardinalis* accessions. (TIF)

S5 Fig The timing and direction on introgression between *M. lewisii* and *M. cardinalis*. Top row: D_FOIL_ statistics using the phylogeny (((Northern *M*. *lewisii*, Sierrran *M*. *lewisii)*,*(M*. *cardinalis*, *M*. *verbenaceus*)),*M*.*bicolor*). A. Boxplots show distributions of D_FOIL_ statistics using all pairwise combinations of *M*. *lewisii* and *M*. *cardinalis*. The combination of positive values near 0.25 for D_FO_ and D_IL_ and near-zero values of D_FI_ and D_OL_ are evidence that the primary genome-wide signature of introgression is between the ancestral *M*. *lewisii* population and *M*. *cardinalis*. For a full explanation of D_FOIL_ statistics, see Pease *et al* (2015). B. Phylogeny summarizing introgression inferred from D_FOIL_. Bottom row: D2 test 1for nuclear introgression being primarily in the direction of *M*. *lewisii* into *M*. *cardinalis*. D2 tests for a difference in coalescence times for gene trees that traverse either the species tree or the introgression tree on a three-species phylogeny (Hahn & Pease, 2019). (C) The two alternatives for the direction of introgression and the expected *M*. *lewisii-M*. *verbenaceus* divergence times (stars) at genes following the species tree (orange) or resulting from introgression (purple). (D) *d*_*xy*_ between Sierran *M*. *lewisii* and *M*. *verbenaceus* at genes supporting the lew-card introgression tree (purple) is not significantly less than *d*_*xy*_ at genes supporting the species tree (orange; two-tailed two sample t-test). **References:** Pease JB, Hahn MW. Detection and polarization of introgression in a five-taxon phylogeny. Syst Biol. 2015;64: 651–662. doi:10.1093/sysbio/syv023; Hahn MW, Hibbins MS. A three-sample test for introgression. Mol Biol Evol. 2019;36: 2878–2882. doi:10.1093/molbev/msz178
(TIF)

S6 Fig Recombination across *M. cardinalis* chromosomal scaffolds. Genetically-mapped gene-targeted capture markers (Nelson *et al*, 2020) are plotted as black circles at their physical (x-axis) and genetic (y-axis) positions, while crosses at x = 0 and the solid line show the density of all targeted capture regions (this study) on each chromosome. v1.92 genome contigs (www.mimubase.org) were ordered and oriented based on the genetic map and local cM/Mbp recombination rates calculated. **References:** Nelson TC, Muir CD, Stathos AM, Vanderpool DD, Anderson K, Angert AL, et al. Quantitative trait locus mapping reveals an independent genetic basis for joint divergence in leaf function, life-history, and floral traits between scarlet monkeyflower (*Mimulus cardinalis*) populations. bioRxiv 2020;101: e02924–35. doi:10.1101/2020.08.16.252916.
(TIF)

S7 Fig TWISST topology weighting on *M. cardinalis* chromosome 1. Topology weights are plotted as in Fig 3 in the main text.
(TIF)

S8 Fig TWISST topology weighting on *M. cardinalis* chromosome 2. Topology weights are plotted as in Fig 3 in the main text.
(TIF)

S9 Fig TWISST topology weighting on *M. cardinalis* chromosome 3. Topology weights are plotted as in Fig 3 in the main text.
(TIF)

S10 Fig TWISST topology weighting on *M. cardinalis* chromosome 4. Topology weights are plotted as in Fig 3 in the main text.
(TIF)

S11 Fig TWISST topology weighting on *M. cardinalis* chromosome 5. Topology weights are plotted as in Fig 3 in the main text.
(TIF)

S12 Fig TWISST topology weighting on *M. cardinalis* chromosome 6. Topology weights are plotted as in Fig 3 in the main text.
(TIF)

S13 Fig TWISST topology weighting on *M. cardinalis* chromosome 7. Topology weights are plotted as in Fig 3 in the main text.
(TIF)

S14 Fig TWISST topology weighting on *M. cardinalis* chromosome 8. Topology weights are plotted as in Fig 3 in the main text.
(TIF)

S15 Fig Rooted maximum likelihood topology from all genome-wide variable sites. Branch supports are ultrafast bootstrap support from IQ-TREE.
(TIF)

S16 Fig Maximum likelihood topology using variable sites from genes in regions of recombination rate ≥ 5 cM/Mbp. Branch supports are ultrafast bootstrap support from IQ-TREE.
(TIF)

S17 Fig Unrooted maximum likelihood chloroplast tree. Branch supports are ultrafast bootstrap support from IQ-TREE.
(TIF)

S18 Fig Unrooted maximum likelihood topology from all (nuclear) genome-wide variable sites. Branch supports are ultrafast bootstrap support from IQ-TREE.
(TIF)

S1 Table Collection site information for accessions used in this study. With the exception of the Vickery accession of *M*. *rupestris*, all sequenced individuals were wild-collected as seeds and greenhouse-grown for tissue collection. The *M*. *bicolor* sample was pooled wild-collected DNA from a large population in the center of the species range. A text version of this table is available at https://github.com/thomnelson/MimulusPhylogenomics/blob/main/Data/lewcardgroup_collectiontable_2021-02-06.csv.
(XLSX)
