# Supplementary figures and images for: Ancient and recent introgression shape the evolutionary history of pollinator adaptation and speciation in a model monkeyflower radiation (Mimulus section Erythranthe)
Source: PLoS Genet. 2021 Feb 22;17(2):e1009095. doi: 10.1371/journal.pgen.1009095 (PMC7951852; doi:10.1371/journal.pgen.1009095)

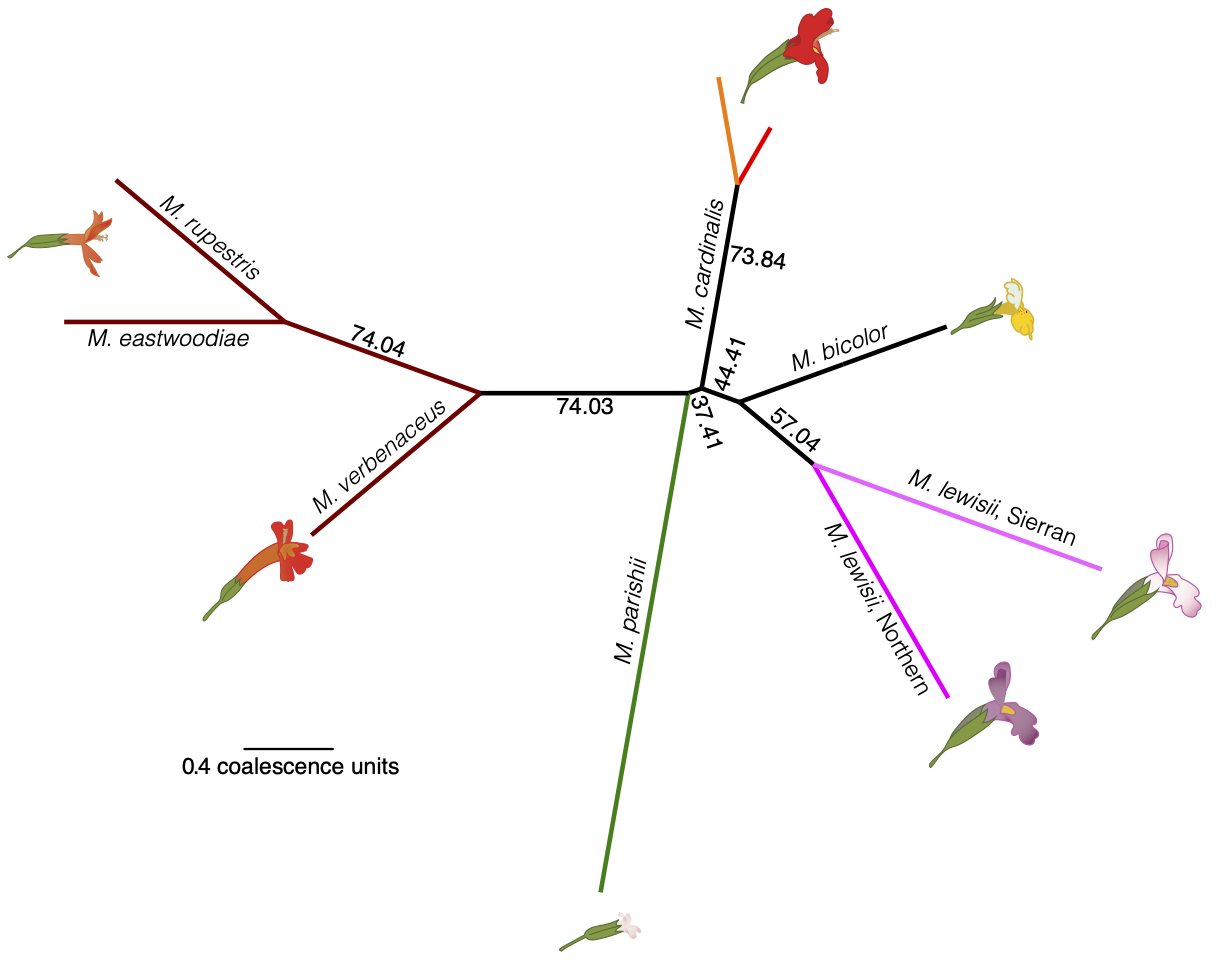

Supplement: S1 Fig — Lengths of internal branches are in coalescence units, as are external branches for species with >1 sample (M. lewisii, M. cardinalis, M. parishii). M. cardinalis samples are split into the Arizona clade (orange) and the California clade (red). Quartet scores for each internal branch are shown; all internal branches have local posterior probabilities of 1. (TIF) [file pgen.1009095.s001.tif]

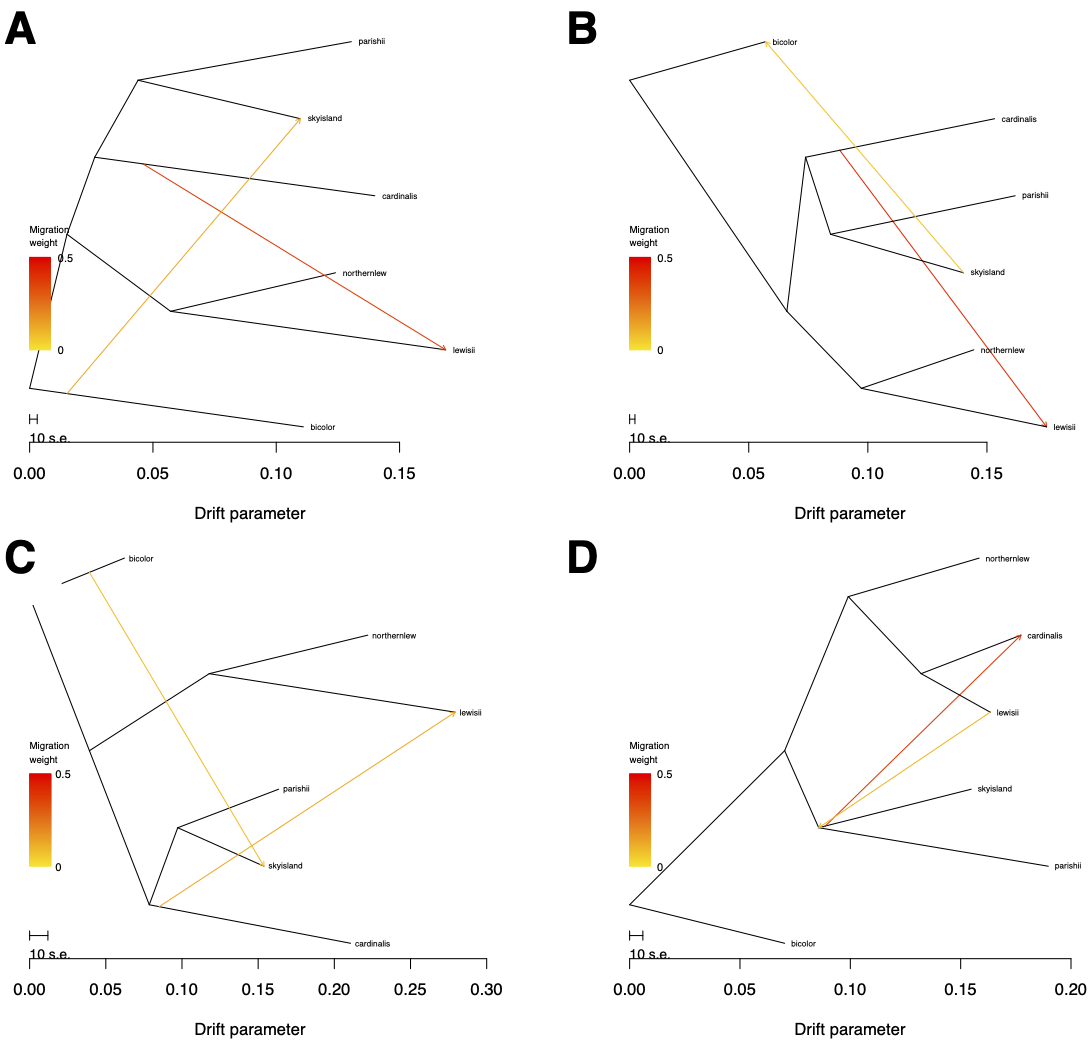

Supplement: S2 Fig — All plots use data pruned for minor allele frequencies ≥ 0.05 and tree inference with two migration edges. A. Using all available SNPs recapitulates the species relationships inferred with IQ-TREE and ASTRAL. The major migration edge corresponds to the recent introgression event from M. cardinalis into M. lewisii in the Sierra Nevada Range. B. Data pruned to exclude SNPs in close physical proximity (100 bp). C. Extended pruning to 1000 bp. D. Using LD-based pruning of SNPs with pairwise R2 ≥ 0.50. (TIF) [file pgen.1009095.s002.tif]

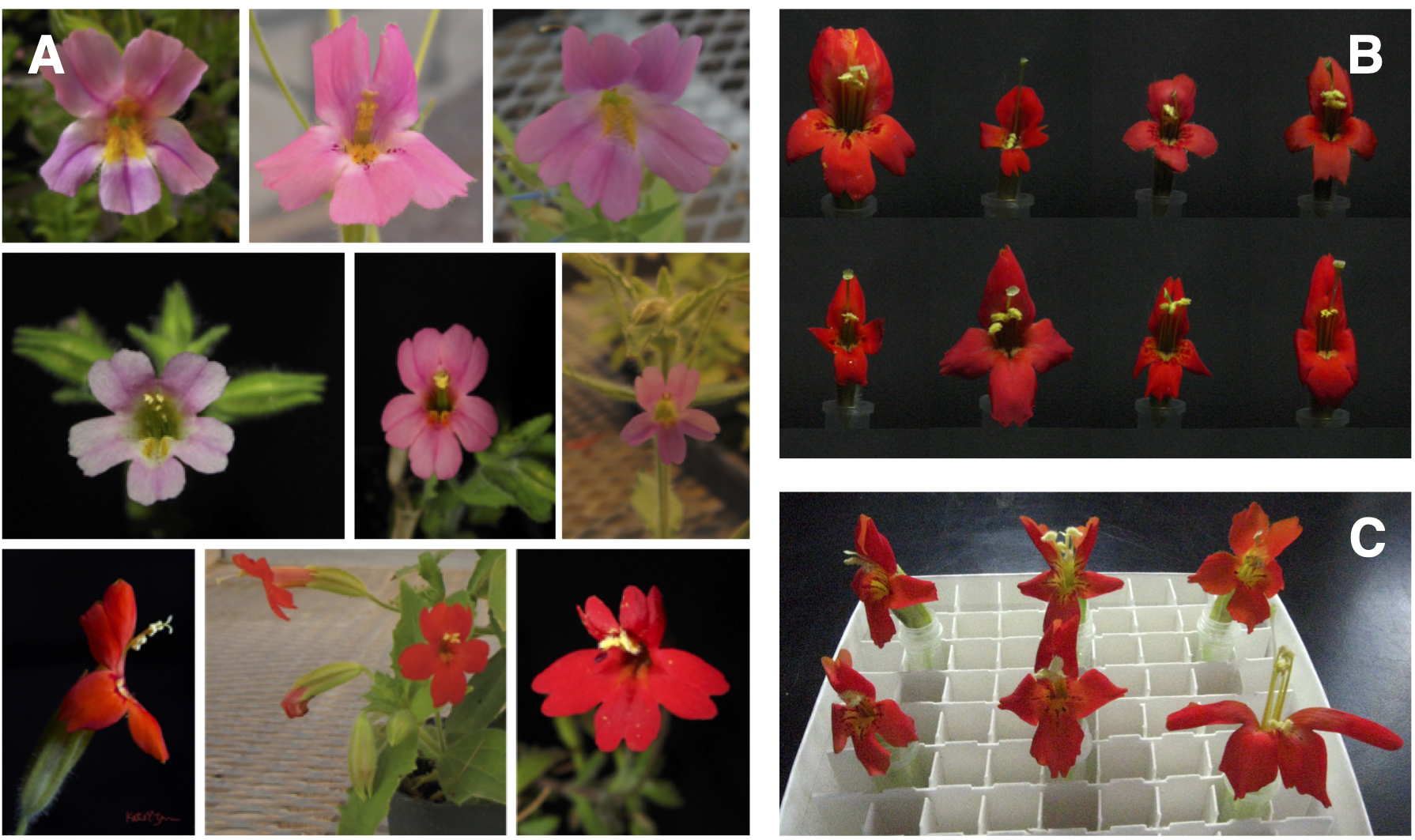

Supplement: S3 Fig — A. Mimulus section Erythranthe species (represented by reference inbred lines) and F1 hybrids. Top row: M. lewisii, M. lewisii x M. cardinalis F1, M. lewisii x M. rupestris F1. Middle row: M. parishii, M. parishii x M. cardinalis F1, M. parishii x M. rupestris F1. Bottom row: M. cardinalis, M. rupestris x M. cardinalis F1, M. rupestris. B. M. rupestris x M. cardinalis F2 hybrids. C. M. verbenaceus x M. cardinalis F2 hybrids. (TIF) [file pgen.1009095.s003.tif]

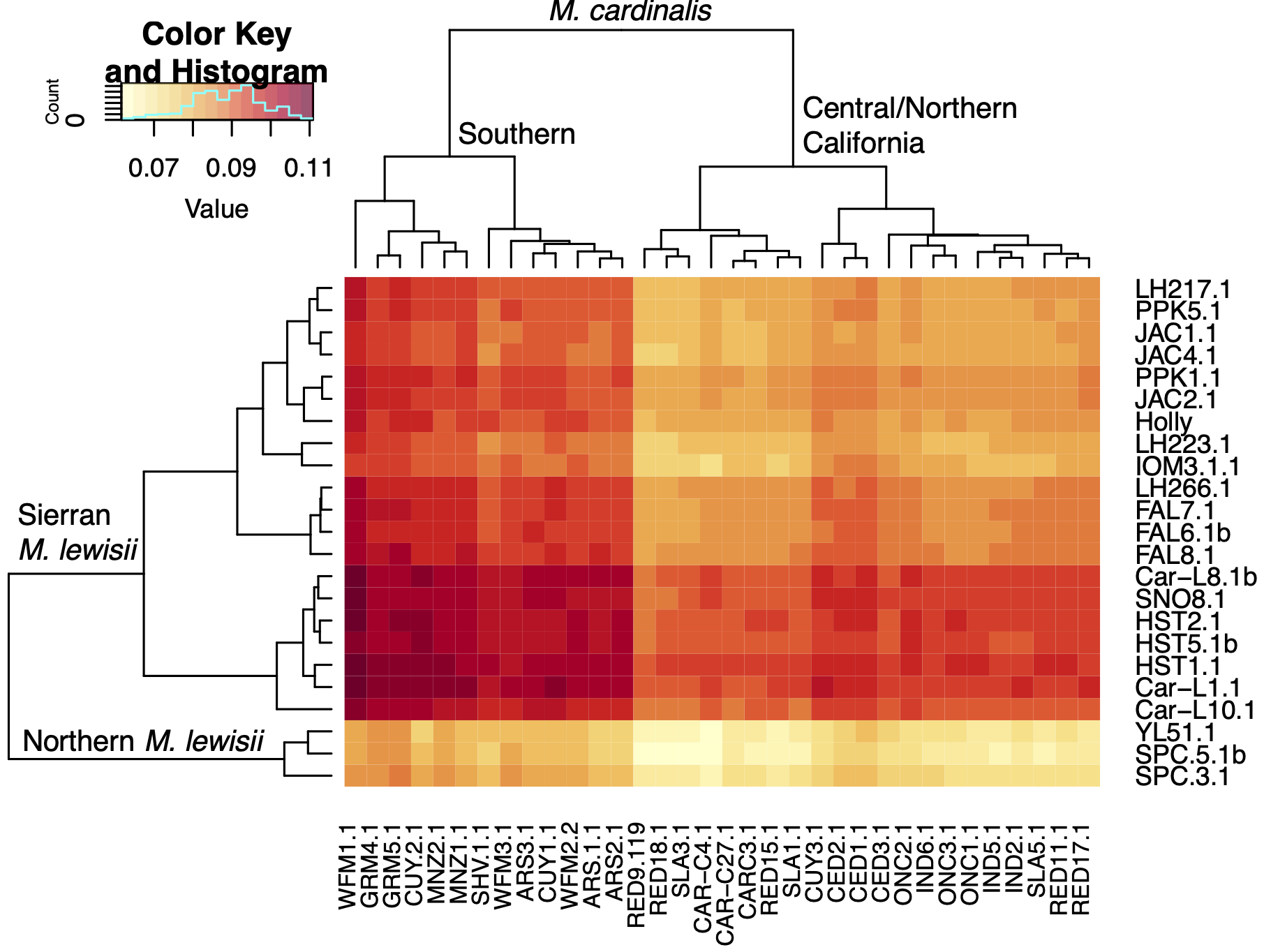

Supplement: S4 Fig — (TIF) [file pgen.1009095.s004.tif]

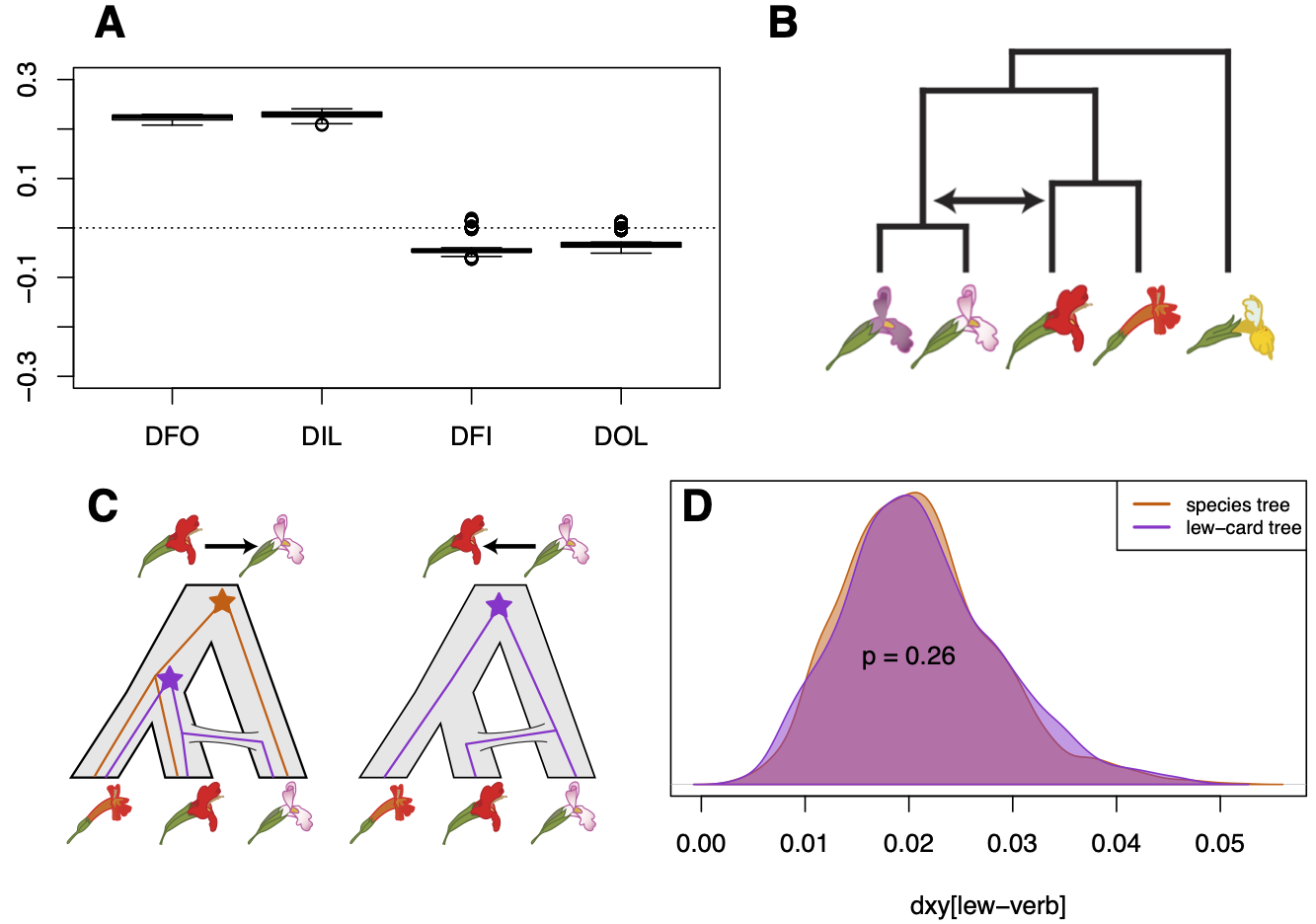

Supplement: S5 Fig — Top row: DFOIL statistics using the phylogeny (((Northern M. lewisii, Sierrran M. lewisii),(M. cardinalis, M. verbenaceus)),M.bicolor). A. Boxplots show distributions of DFOIL statistics using all pairwise combinations of M. lewisii and M. cardinalis. The combination of positive values near 0.25 for DFO and DIL and near-zero values of DFI and DOL are evidence that the primary genome-wide signature of introgression is between the ancestral M. lewisii population and M. cardinalis. For a full explanation of DFOIL statistics, see Pease et al (2015). B. Phylogeny summarizing introgression inferred from DFOIL. Bottom row: D2 test 1for nuclear introgression being primarily in the direction of M. lewisii into M. cardinalis. D2 tests for a difference in coalescence times for gene trees that traverse either the species tree or the introgression tree on a three-species phylogeny (Hahn & Pease, 2019). (C) The two alternatives for the direction of introgression and the expected M. lewisii-M. verbenaceus divergence times (stars) at genes following the species tree (orange) or resulting from introgression (purple). (D) dxy between Sierran M. lewisii and M. verbenaceus at genes supporting the lew-card introgression tree (purple) is not significantly less than dxy at genes supporting the species tree (orange; two-tailed two sample t-test). References: Pease JB, Hahn MW. Detection and polarization of introgression in a five-taxon phylogeny. Syst Biol. 2015;64: 651–662. doi:10.1093/sysbio/syv023; Hahn MW, Hibbins MS. A three-sample test for introgression. Mol Biol Evol. 2019;36: 2878–2882. doi:10.1093/molbev/msz178 (TIF) [file pgen.1009095.s005.tif]

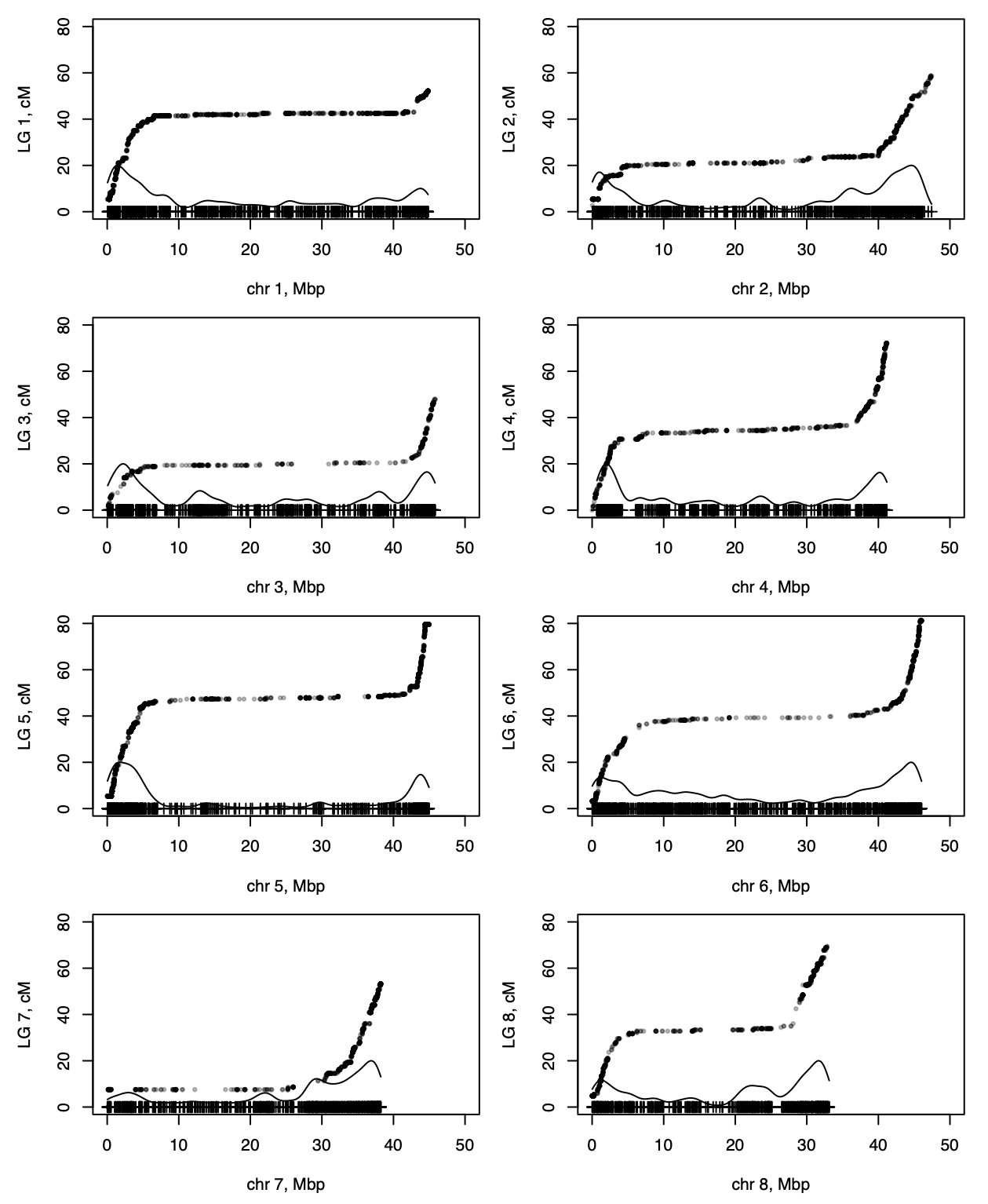

Supplement: S6 Fig — Genetically-mapped gene-targeted capture markers (Nelson et al, 2020) are plotted as black circles at their physical (x-axis) and genetic (y-axis) positions, while crosses at x = 0 and the solid line show the density of all targeted capture regions (this study) on each chromosome. v1.92 genome contigs (www.mimubase.org) were ordered and oriented based on the genetic map and local cM/Mbp recombination rates calculated. References: Nelson TC, Muir CD, Stathos AM, Vanderpool DD, Anderson K, Angert AL, et al. Quantitative trait locus mapping reveals an independent genetic basis for joint divergence in leaf function, life-history, and floral traits between scarlet monkeyflower (Mimulus cardinalis) populations. bioRxiv 2020;101: e02924–35. doi:10.1101/2020.08.16.252916. (TIF) [file pgen.1009095.s006.tif]

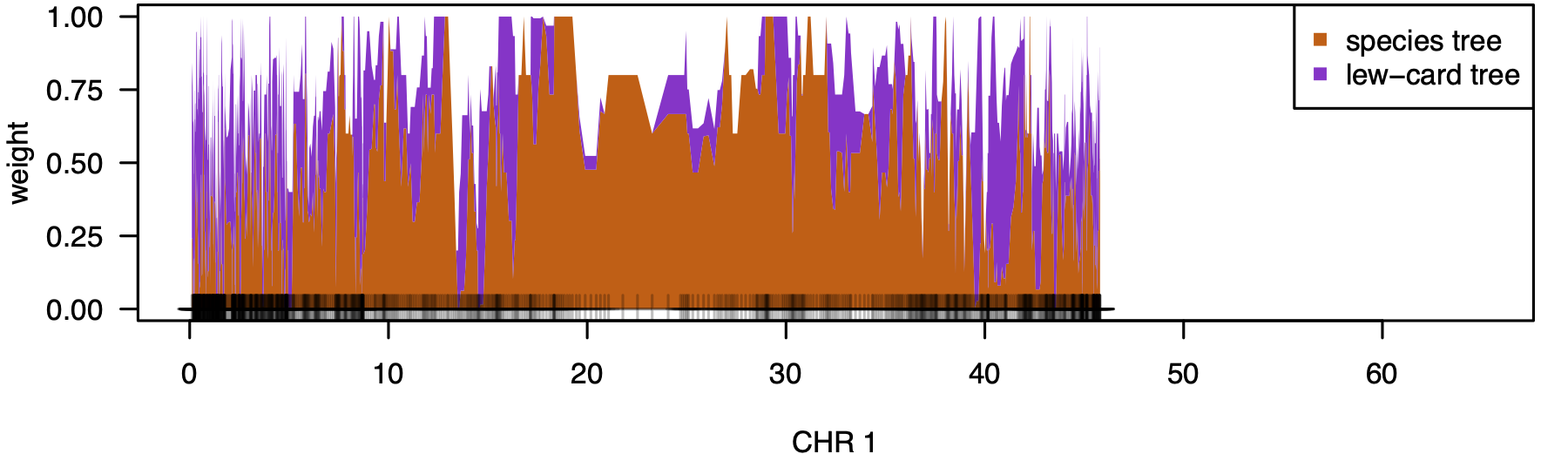

Supplement: S7 Fig — Topology weights are plotted as in Fig 3 in the main text. (TIF) [file pgen.1009095.s007.tif]

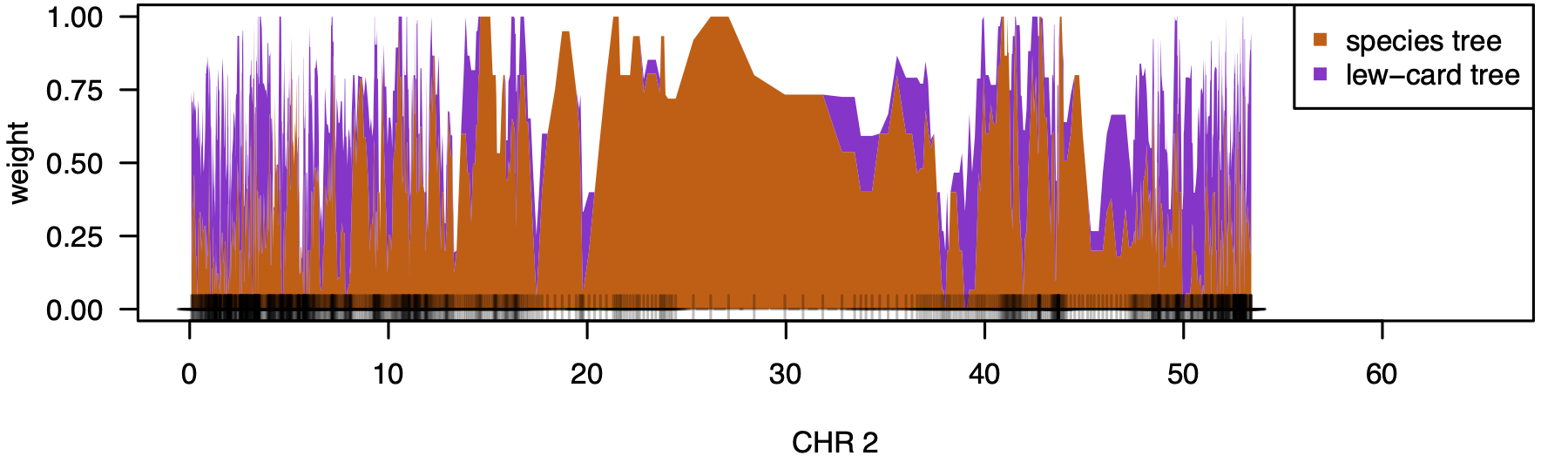

Supplement: S8 Fig — Topology weights are plotted as in Fig 3 in the main text. (TIF) [file pgen.1009095.s008.tif]

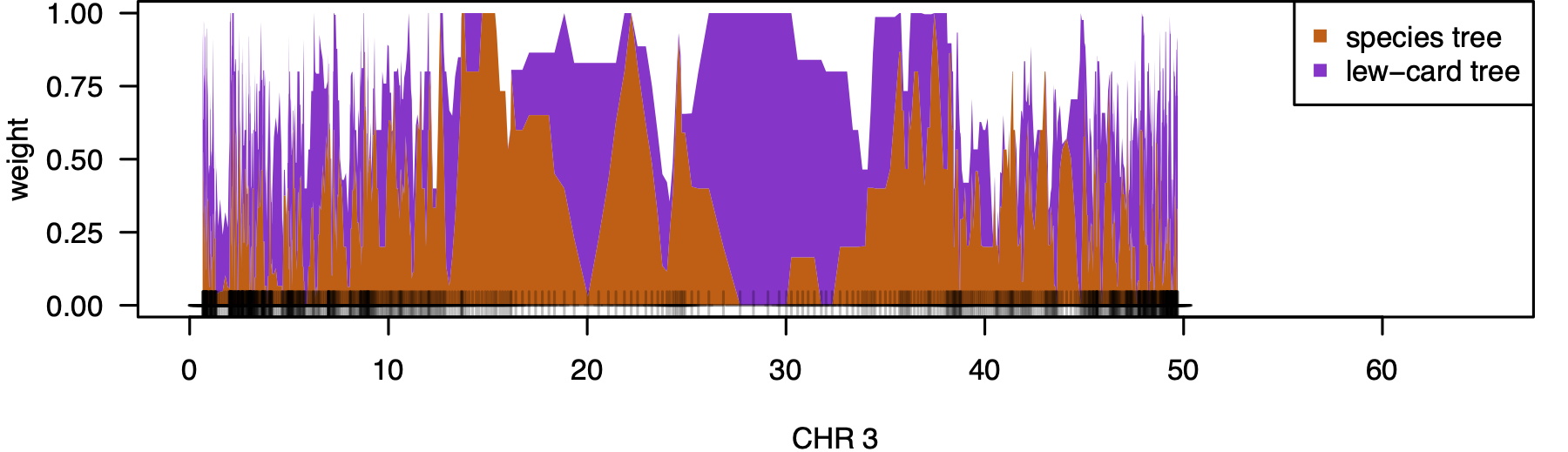

Supplement: S9 Fig — Topology weights are plotted as in Fig 3 in the main text. (TIF) [file pgen.1009095.s009.tif]

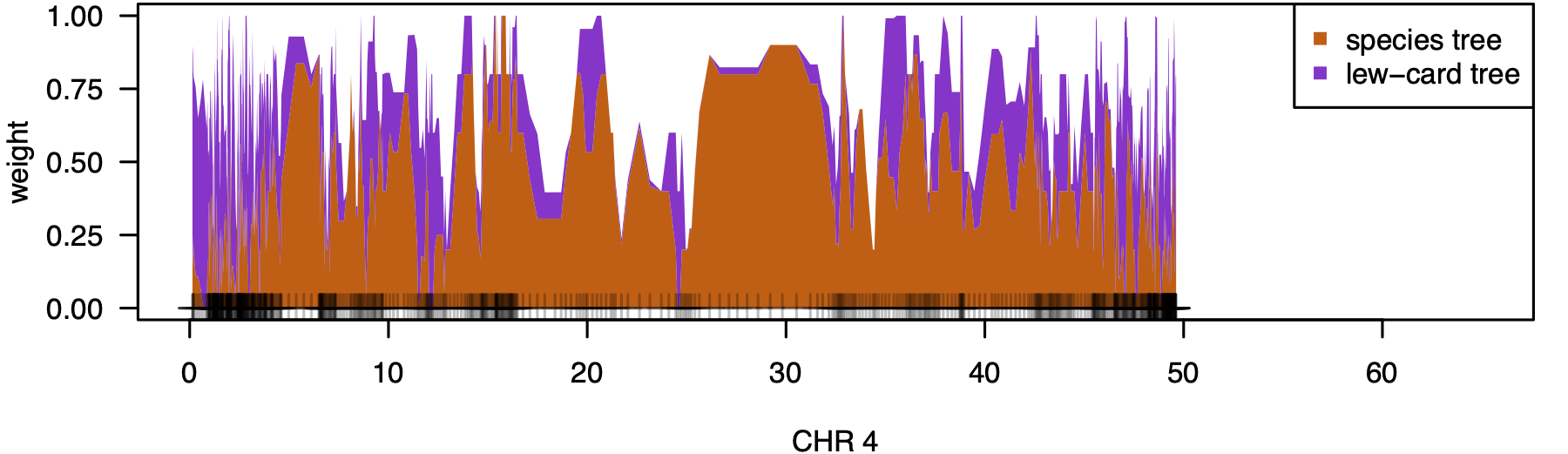

Supplement: S10 Fig — Topology weights are plotted as in Fig 3 in the main text. (TIF) [file pgen.1009095.s010.tif]

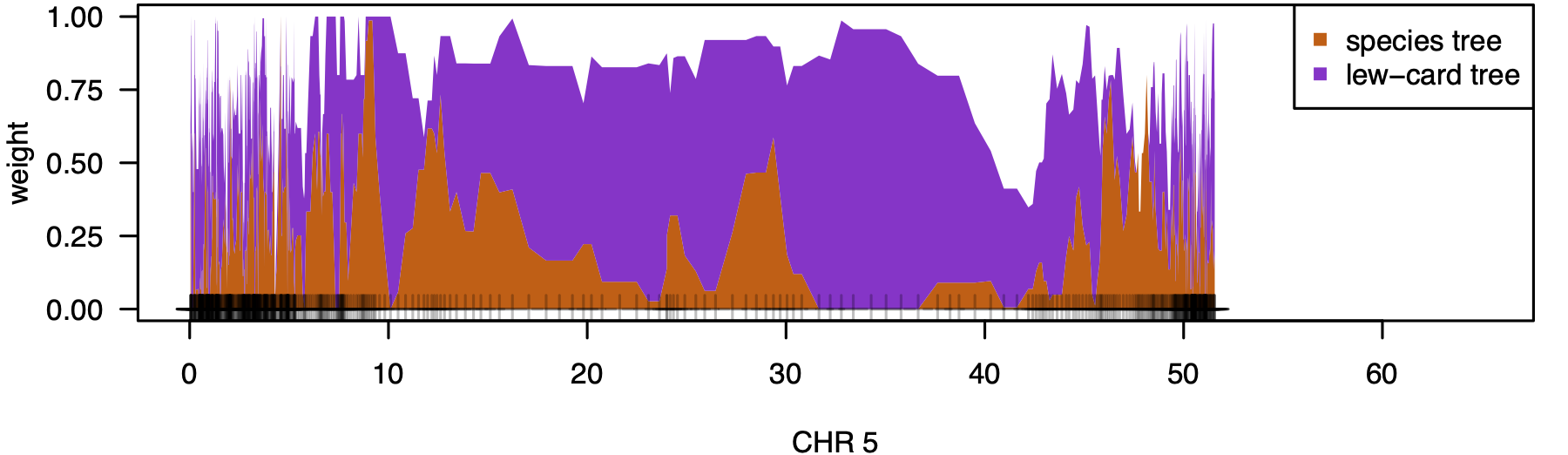

Supplement: S11 Fig — Topology weights are plotted as in Fig 3 in the main text. (TIF) [file pgen.1009095.s011.tif]

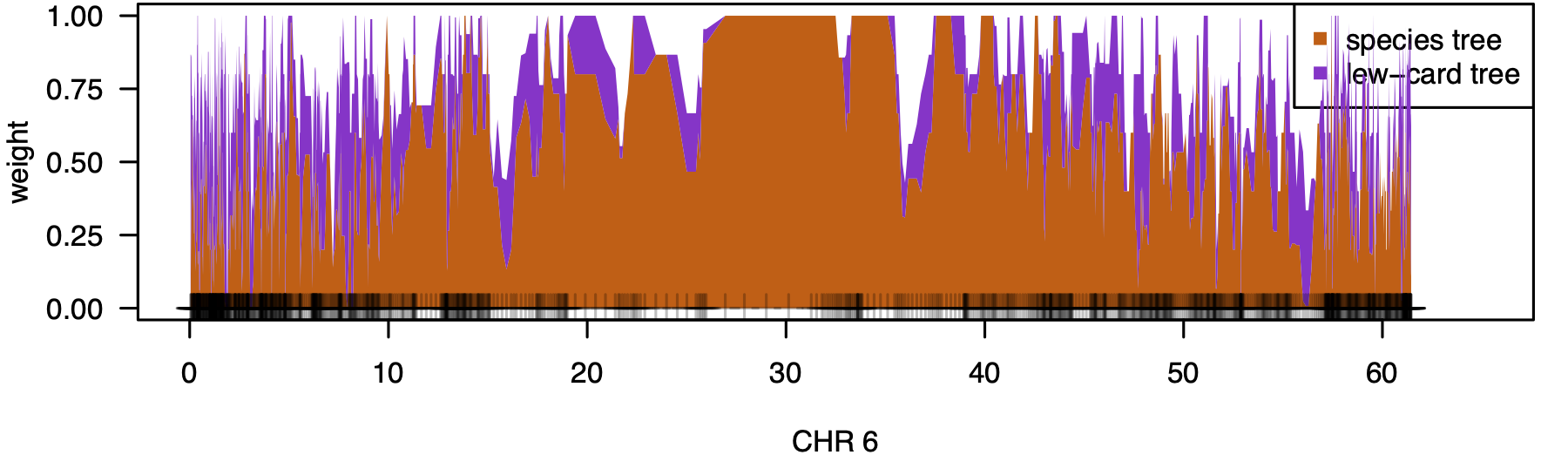

Supplement: S12 Fig — Topology weights are plotted as in Fig 3 in the main text. (TIF) [file pgen.1009095.s012.tif]

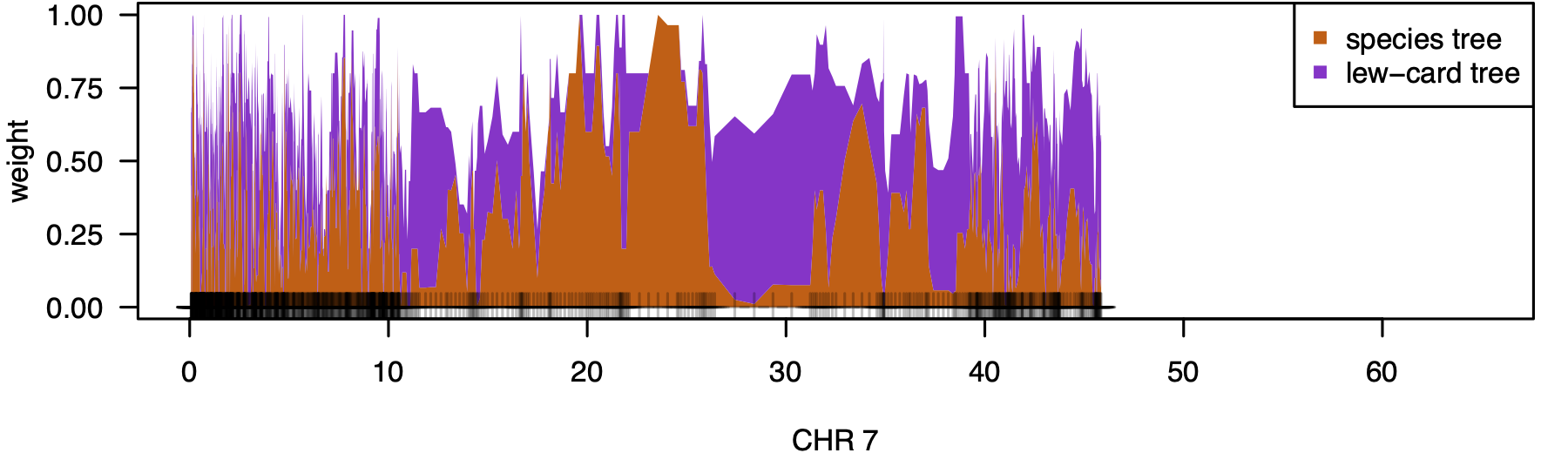

Supplement: S13 Fig — Topology weights are plotted as in Fig 3 in the main text. (TIF) [file pgen.1009095.s013.tif]

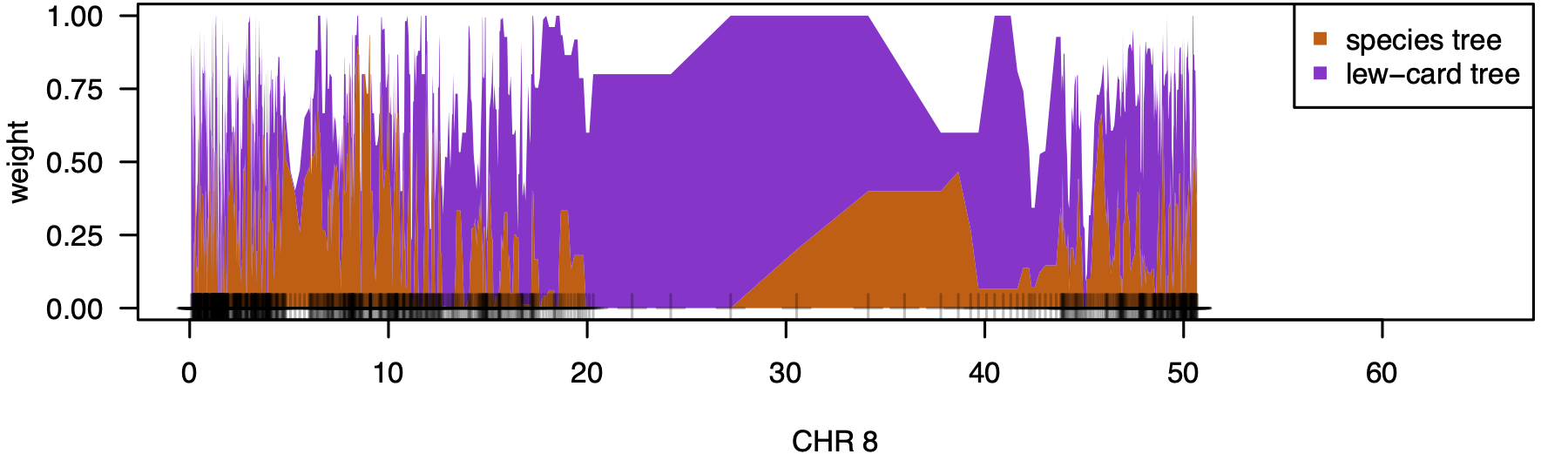

Supplement: S14 Fig — Topology weights are plotted as in Fig 3 in the main text. (TIF) [file pgen.1009095.s014.tif]

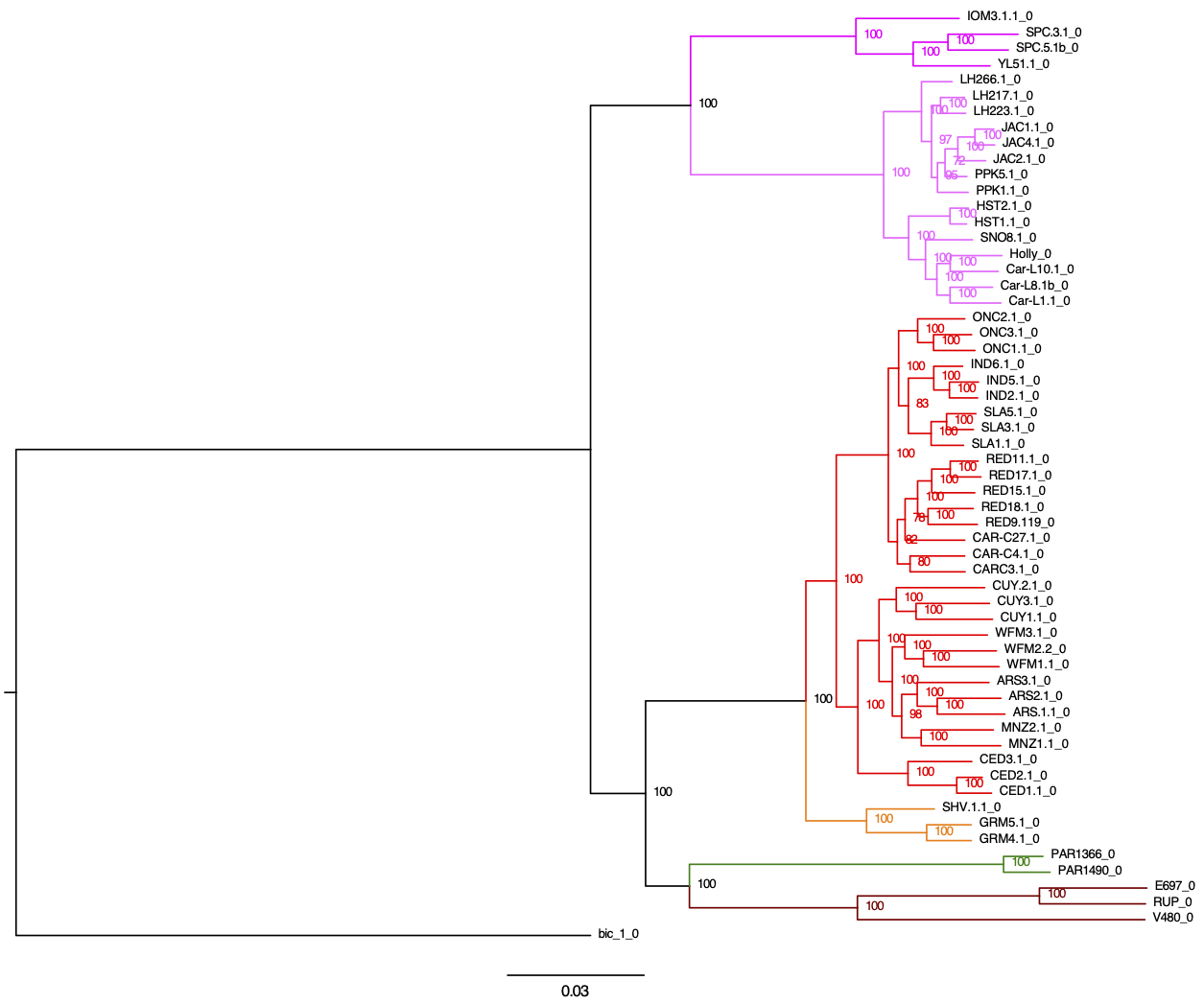

Supplement: S15 Fig — Branch supports are ultrafast bootstrap support from IQ-TREE. (TIF) [file pgen.1009095.s015.tif]

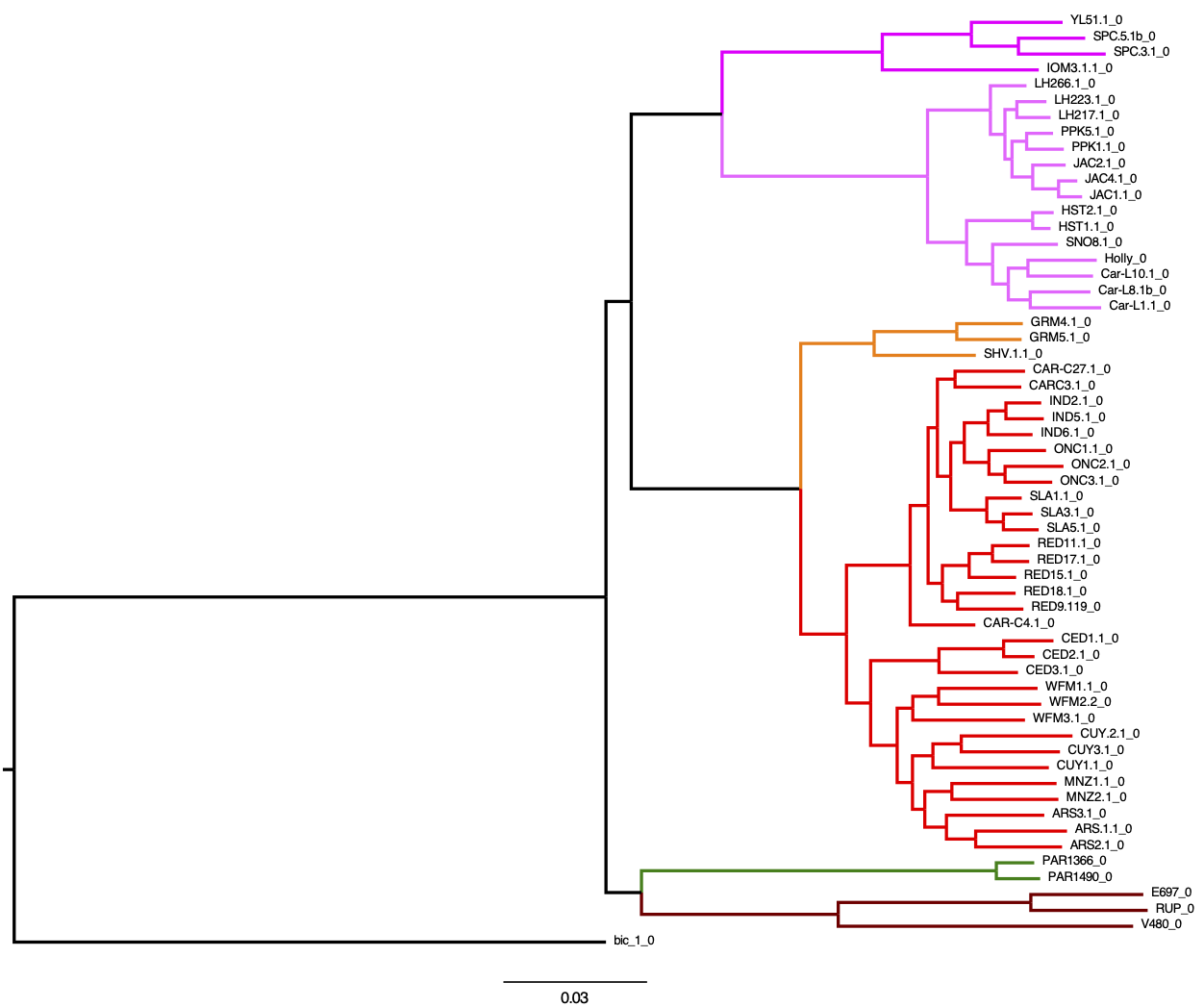

Supplement: S16 Fig — Branch supports are ultrafast bootstrap support from IQ-TREE. (TIF) [file pgen.1009095.s016.tif]

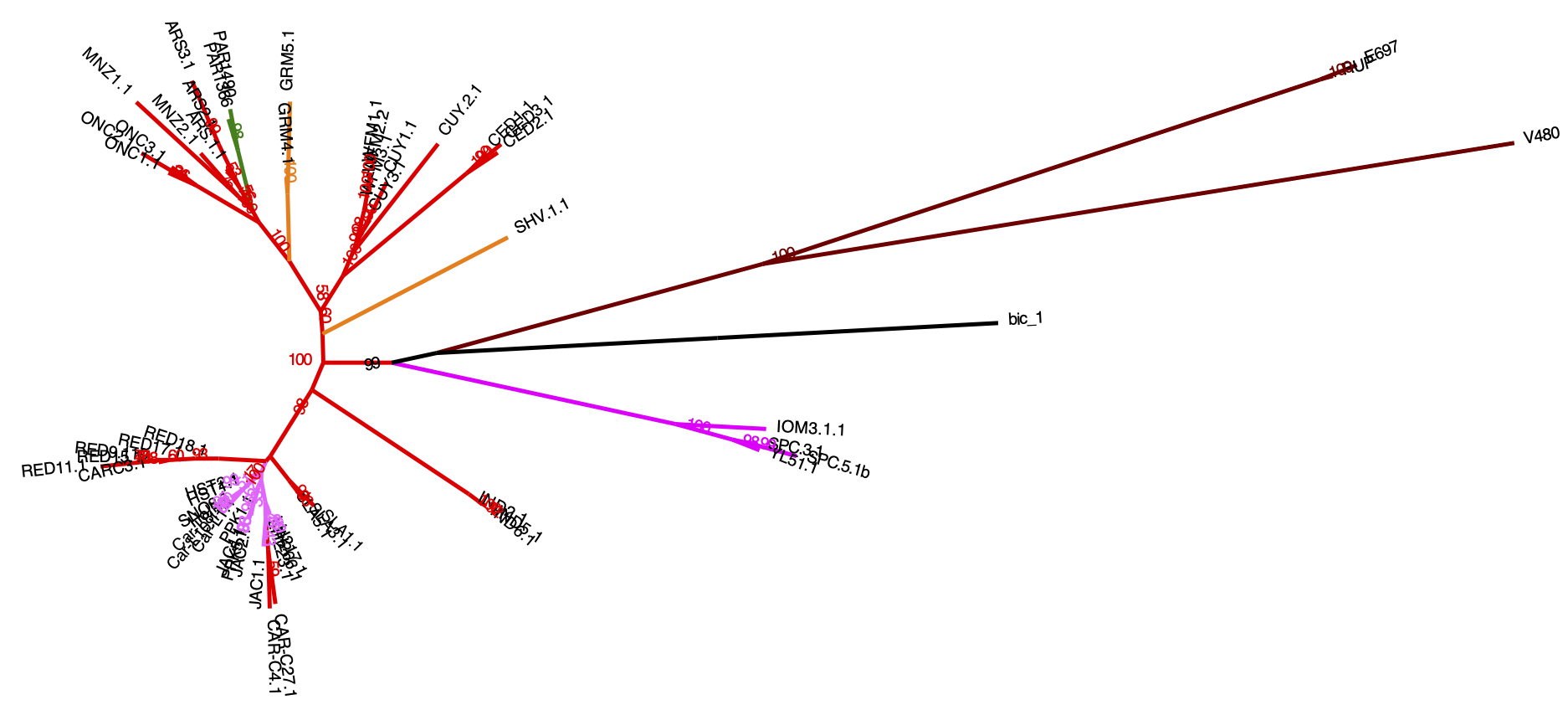

Supplement: S17 Fig — Branch supports are ultrafast bootstrap support from IQ-TREE. (TIF) [file pgen.1009095.s017.tif]

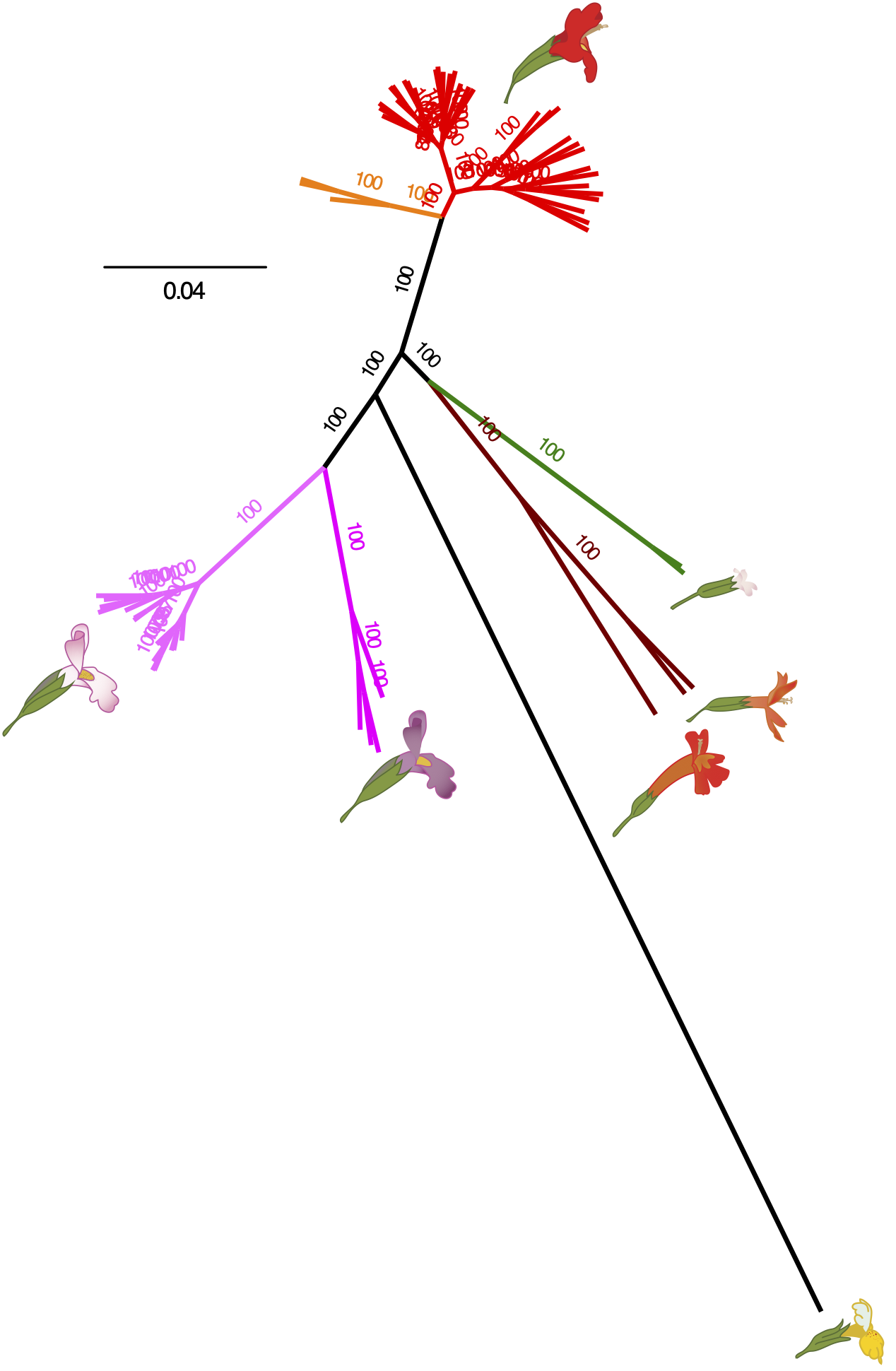

Supplement: S18 Fig — Branch supports are ultrafast bootstrap support from IQ-TREE. (TIF) [file pgen.1009095.s018.tif]
